# An unbroken network of interactions connecting flagellin domains is required for motility in viscous environments

**DOI:** 10.1371/journal.ppat.1010979

**Published:** 2023-05-30

**Authors:** Marko Nedeljković, Mark A. B. Kreutzberger, Sandra Postel, Daniel Bonsor, Yingying Xing, Neil Jacob, William J. Schuler, Edward H. Egelman, Eric J. Sundberg

**Affiliations:** 1 Department of Biochemistry, Emory University School of Medicine, Atlanta, Georgia, United States of America; 2 Department of Biochemistry and Molecular Genetics, University of Virginia School of Medicine, Charlottesville, Virginia, United States of America; 3 Institute of Human Virology, University of Maryland School of Medicine, Baltimore, Maryland, United States of America; 4 School of Life Science and Technology, China Pharmaceutical University, Nanjing, China; Children’s Hospital Boston, UNITED STATES

## Abstract

In its simplest form, bacterial flagellar filaments are composed of flagellin proteins with just two helical inner domains, which together comprise the filament core. Although this minimal filament is sufficient to provide motility in many flagellated bacteria, most bacteria produce flagella composed of flagellin proteins with one or more outer domains arranged in a variety of supramolecular architectures radiating from the inner core. Flagellin outer domains are known to be involved in adhesion, proteolysis and immune evasion but have not been thought to be required for motility. Here we show that in the *Pseudomonas aeruginosa* PAO1 strain, a bacterium that forms a ridged filament with a dimerization of its flagellin outer domains, motility is categorically dependent on these flagellin outer domains. Moreover, a comprehensive network of intermolecular interactions connecting the inner domains to the outer domains, the outer domains to one another, and the outer domains back to the inner domain filament core, is required for motility. This inter-domain connectivity confers PAO1 flagella with increased stability, essential for its motility in viscous environments. Additionally, we find that such ridged flagellar filaments are not unique to *Pseudomonas* but are, instead, present throughout diverse bacterial phyla.

## Author summary

Opportunistic bacteria like *Pseudomonas aeruginosa* use flagella to move freely through the environment, adhere to surfaces and infect the host. The most prominent component of the flagellum is a hollow filament made of thousands of copies of the protein flagellin, or FliC. The inner part of the filament, the core, is structurally the same in all flagellated bacteria, while surface-exposed flagellin domains differ significantly, and their exact role is not fully understood. Here we present a new type of filament organization in which outer domains establish extensive contacts with the core and dimerize along the axis resulting in a ridged filament type. By studying the effects of mutations on bacterial swimming, we found that outer domains of the ridged filaments of *Pseudomonas* are indispensable for swimming, unlike the outer domains of *Salmonella*. Certain contacts between outer domains themselves and outer domains and the core allow bacteria to swim efficiently through porous or viscous environments. We found that this type of filament organization is present in many other bacterial species not closely related to *P*. *aeruginosa*, suggesting that this type of filament architecture could be advantageous in colonization.

## Introduction

The bacterial flagellum is a complex and dynamic nanomachine that propels bacteria through liquids. In pathogenic species, motility provided by flagella is critical for host colonization and infection, and flagellar filaments are recognized as important virulence factors involved in adherence, toxin delivery, biofilm formation and activation of innate immunity [1–8]. The number of bacterial flagella attached to the cell body varies among species, from one, as in *Pseudomonas aeruginosa*, to many, as in *Escherichia coli* and *Salmonella enterica* serovar Typhimurium [9]. Each flagellum consists of a membrane-embedded basal body that functions as a motor, as well as a hook and a long filament [10,11]. Torque generated by the basal body is transferred by the hook to the filament, rotation of which provides a thrust that propels bacteria through liquid.

The flagellar filament, a tubular structure composed of thousands of copies of the protein flagellin (FliC in many bacteria), exhibits helical symmetry in its straight non-motile forms [12] and is supercoiled with a three-dimensional waveform in motile bacteria. In these straight forms, one FliC molecule is copied eleven times along the screw axis, making two turns, before it reaches the position above this starting position. Such an arrangement results in stacks of FliC along the filament axis called protofilaments. In a wildtype filament, however, the helical symmetry is subtly broken allowing for supercoiling to occur. The filament is capped at its distal end by an oligomeric stool-like structure comprised of five or six copies of the protein FliD [13,14]. FliC molecules are synthesized in the cytoplasm and exported through the flagellar type III secretion system at the base of the flagellum [15]. Since the filament extends from its distal end, FliC is transported through the central channel of the filament, which measures approximately 25 Å in diameter. Thus, FliC must be at least partially unfolded while passing through the channel and, once it reaches the FliD cap, attains the correct structure and is positioned at the tip of the filament [15,16]. This process is still not fully elucidated, but involves direct interactions between incoming FliC subunits and the oligomeric FliD cap in a species-specific manner [17].

The flagellar system of *Salmonella* has been studied as the archetypal model of bacterial flagella for decades. The first flagellin protein structure was from that of *S*. Typhimurium [18], revealing a boomerang-shaped molecule with four domains—the D0 and D1 inner domains that are predominantly α-helical and comprise the filament core, and the propeller-shaped D2 and D3 outer domains that protrude from the filament at an angle of approximately 90 degrees to the filament axis [18,19]. In *S*. Typhimurium flagellar filaments, the outer domains are splayed outward from the filament core such that no extensive interactions are made between the D2 and D3 domains of neighboring subunits along the protofilament [18,20]. This is corroborated by mutational studies that have shown that large truncations of the *S*. Typhimurium outer domains are tolerated without abolishing motility [21]. Recently, structures of flagellins and filaments from other species have become available, confirming that the inner core, composed of D0 and D1 domains, is structurally conserved, while there is extensive variability in the sequence, length, structure and arrangement of outer domains in FliC [22–24]. In some motile bacteria, the outer domains are completely absent, as observed for *Bacillus subtilis* and *Kurthia* sp. [25,26]. A recent series of high-resolution structures of so-called complex filaments showed that outer domains can oligomerize to generate helical threads or mesh-like sheath structures that serve to stabilize the filament form [24]. Thus, flagellated motile bacteria all share a highly conserved inner core decorated by a widely divergent collection of outer domains.

Until recently, technical challenges limited structural studies to straight flagellar filaments that exhibit helical symmetry. Some of the first filament structures were derived from *Salmonella* mutants that were flagellated but immotile due to the fact that mutations of flagellin in the D1 domains locked it in one of the two conformations—having all left- (L) or all right- (R) handed protofilaments, resulting in straight filaments [27]. Equivalent mutations produced the same effect in other species, including *B*. *subtilis*, *P*. *aeruginosa* and *Campylobacter jejuni* [23,25], and it was postulated that both protofilament forms were present in the wild type, with different combinations of L- and R-handed protofilaments determining the macroscopic supercoil of the filament and, consequently, swimming speed. Recent advances in EM cameras and data processing enabled us to study filaments in their native supercoiled state and showed that filaments consist of 11 distinct protofilament conformations instead of L- and R-handed protofilaments [28], which are artefacts of the mutations in their core domains. Outer domains, on the other hand, have not been shown to contribute to motility and, instead, are thought to mediate many of the other roles in which flagella are implicated, such as adherence and biofilm formation. However, the growing catalog of diverse flagellar filament supramolecular architectures suggests that FliC outer domains could, in certain bacteria, play a critical role in motility. The previously reported cryo-EM structures of L- and R-handed filaments from the *P*. *aeruginosa* strain PAO1 revealed that the overall shape of this filament was ridged, unlike the splayed geometry of the *S*. Typhimurium filament [25]. In the former, the D2 and D3 outer domains of FliC adopt an end-on-end compact fold along the filament axis, resulting in ridges along the filament where they are present and clefts along the filament where they are absent. While the D0/D1 core of the L- and R-state mutants from PAO1 were reconstructed at ~ 4.2 Å resolution, it was observed that the outer domains had a pairing or dimerization that introduced a seam (a helical discontinuity) into the structure [25]. The consequence of this was a very low local resolution (>10 Å) for the D2 and D3 domains in the cryo-EM structure. The intermolecular interactions maintaining the ridges, as well as the connections between the ridges and the inner core, were therefore not resolved. In order to obtain a complete model of the PAO1 filament and to study the functional consequences of the “ridged” structural organization, we turned to X-ray crystallography to resolve the structure of the missing D2-D3 domains and, subsequently, performed a cryo-EM reconstruction of the native *P*. *aeruginosa* PAO1 supercoiled filament without imposing helical symmetry. Based on the resulting model, we identified three interfaces that the D2 and D3 outer domains form with one another as well as with the D1 inner domain. Combining genetic tools, phase-contrast light and electron microscopy and swimming motility assays, we showed that these interfaces form a connected network of inter-domain interfaces that are critical not only for the structural integrity of the filament, but also for motility of the bacterium. Using bioinformatic tools, we found that ridged flagellar filaments similar to that in *P*. *aeruginosa* PAO1 are common throughout the bacterial kingdom and that such interconnected filament architectures may provide an advantage to bacteria in generating the thrust needed to swim through liquids of high viscosity.

## Results

### High-resolution X-ray crystal structure of the *P*. *aeruginosa* PAO1 FliC D2 and D3 domains

**Fig 1:**
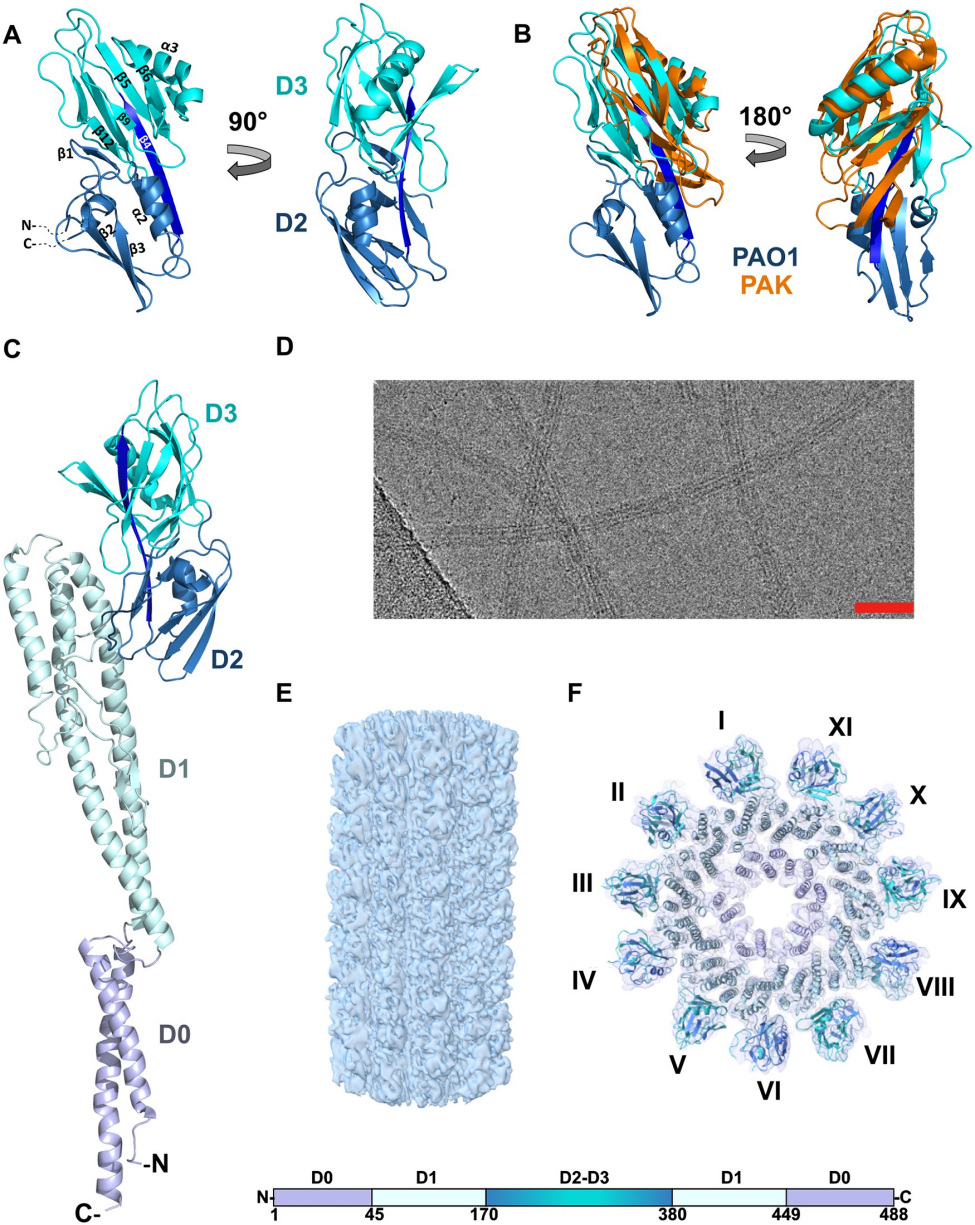
(A) Crystal structure of the FliC D2/D3 domains from *P*. *aeruginosa*. Marine blue—D2, cyan—D3, dark blue—common beta-strand. Alpha-helices and beta-strands are numbered in order of appearance in D2/D3 domains, not in full-length FliC. (B) Superposition of outer domain structures of *P*. *aeruginosa* PAO1 (color code as in A) and PAK (orange) strains. (C) Full-length FliC of PAO1 with schematic representation of FliC protein organization. Colors on the bar correspond to domain colors on the structure. (D) Cryo-electron micrograph of the PAO1 flagellar filaments. The scale bar represents ~400 Å. (E) Cryo-EM reconstruction of the PAO1 flagellar filament with curvature present due to not imposing helical symmetry on the reconstruction. (F) Top-down view of the PAO1 filament model fit into the density map.

Although the cryo-EM structure of the PAO1 filament achieved near-atomic resolution for the inner core of the filament, the resolution of the outer domains was substantially lower, prohibiting even the tracing of the main chain of the D2 and D3 domains [25]. Thus, we expressed and purified the fragment of FliC between residues 178–395 that forms the D2 and D3 domains and crystallized both the native protein and its selenomethionine derivative in the P2 space group with 2 molecules in the asymmetric unit. Employing multi-wavelength anomalous dispersion (MAD) phasing methods, we arrived at a 1.47 Å resolution structure revealing the D2 and D3 domains as a contiguous barrel-like structure composed predominantly of β-strands, with D3 positioned directly above D2 along the filament axis towards the distal end of the filament (Figs 1A, S1A and S1B and S1 Table). While D3 in *S*. Typhimurium is inserted into D2 and they form two separated domains, a different topology of the secondary structural elements in PAO1 FliC results in the D2 and D3 domains folding into a single bilobal structure with two distinct surfaces, the concave surface facing the filament core, and the convex surface exposed to the outside. Starting from the N-terminus, the first β-strand is incorporated into the D3 moiety, before the backbone turns downward, forming one half of D2 (S1C Fig). The sequence between 243 and 353 is entirely a part of D3, after which the polypeptide chain returns to complete the fold of D2. On the concave side of the D2/D3 domain, residues between 233 and 242 belong to the β4-strand that occupies central position spanning both D2 and D3 and together with strands 7, 8, 10 and 11 of D3 and strand 13 of D2 they form an antiparallel β-sheet. The consequence of this organization is the tight packing of D2 and D3 which are oriented along the filament, instead of projecting away from it as in the *S*. Typhimurium.

Two types of FliC proteins are found in *P*. *aeruginosa*: type A as observed in the PAK strain; or type B as seen in *P*. *aeruginosa* PAO1 [29]. The inner domains display high sequence identity, while the outer domains share poor sequence conservation and size. Compared to the previously determined structure of the type A PAK FliC [22], our type B PAO1 FliC outer domains are oriented similarly relative to the inner core. However, the type A D2 domain of PAK is 90 residues smaller and structurally corresponds to the D3 moiety in PAO1 type B FliC (Fig 1B). We then made a composite model of the PAO1 flagellin from the outer domain crystal structure and the previous cryo-EM model of domains D0 and D1 (Fig 1C).

### Cryo-EM reconstruction of native filament

A recent study was able to use cryo-EM to reconstruct, without imposing helical symmetry, the H6 flagellar filament from Enteropathogenic *E*. *coli* O127:H6 with a seam to 4.2 Å. This led to the ability to trace the chain in most of the outer domains, and to show the local seam interactions at 6.7 Å resolution [24]. We used the same method here to reconstruct the PAO1 flagellar filament (Figs 1D–1E, S2, and S3) to 5 Å resolution. Using the composite flagellin model (Fig 1C), we built a model of the full PAO1 flagellar filament and then fit it into the density map (Fig 1F).

**Fig 2:**
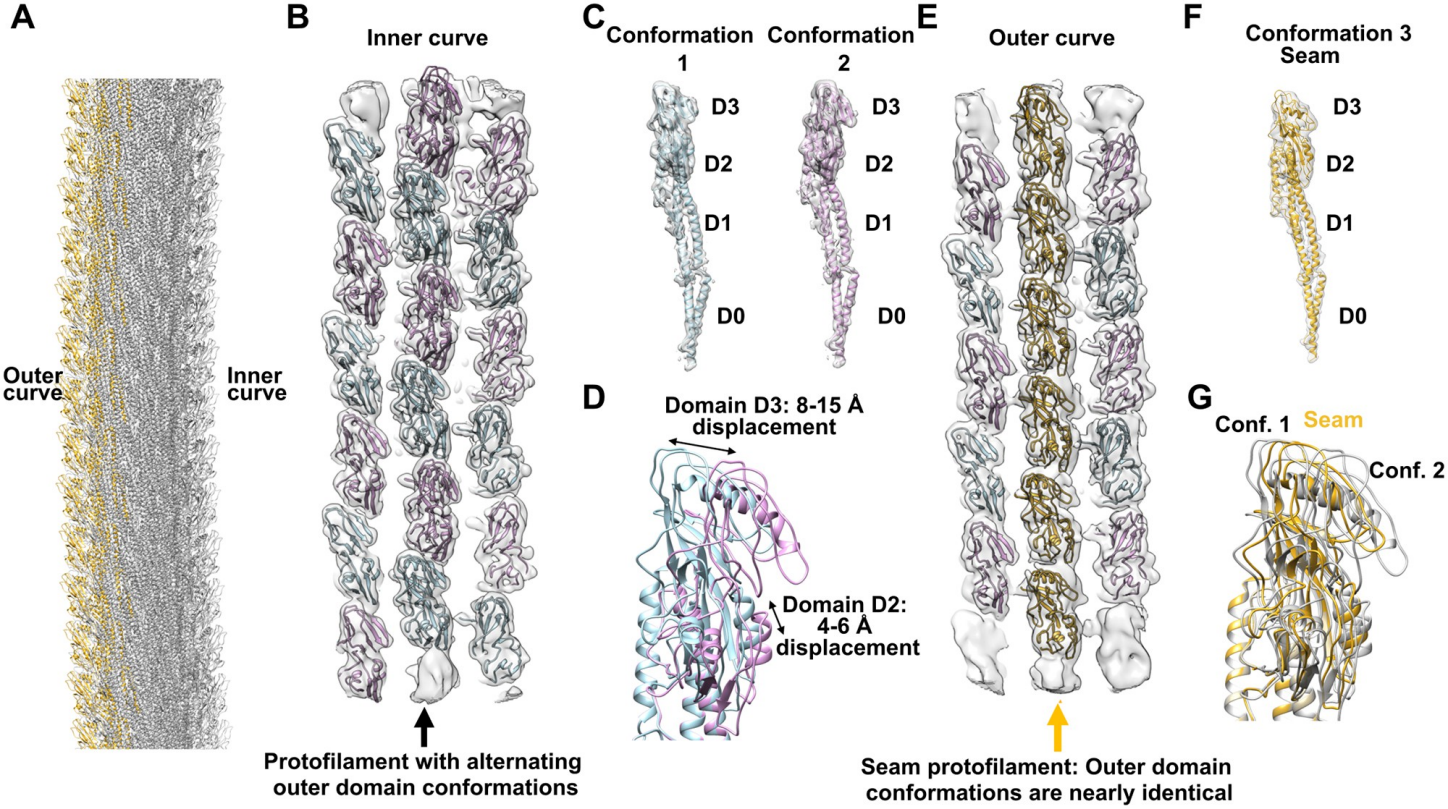
(A) Supercoiled model of the PAO1 flagellar filament showing curvature created by aligning several copies of the filament model as previously described [28]. A single protofilament on the outer curve is colored gold. (B) Cryo-EM density map (grey) and model showing the outer domains for the inner curve protofilaments. Models colored blue and pink have distinct outer domain conformations. (C) Subunits with the two different outer domain conformations observed for 10 PAO1 protofilaments. Conformation 1 (blue) has its outer domain tilted counterclockwise with respect to filament axis while conformation two has its outer domain tilted clockwise. (D) Alignment of conformations 1 and 2 by domains D0 and D1 reveals a displacement of 4–6 Å in domain D2 and 8–15 Å in domain D3 between the two conformations. (E) The outer-most curved protofilaments with a seam protofilament (gold) having subunits in nearly identical conformations. (F) A flagellin subunit from the seam protofilament. (G) Comparison of the outer domain orientations of the conformation 1 and 2 subunits with the seam subunit (conformation 3).

Due to not imposing helical symmetry on our reconstruction there, we observed an inherent curvature in the density map, arising from the supercoiling of the filaments, which was preserved in the refined model we built. This curvature can be seen very easily if multiple copies of the PAO1 filament are aligned end-to-end (Fig 2A) as previously published [28]. Analysis of the outer domain interactions along 10 of the 11 protofilaments revealed a distinct “zig-zag” pattern of outer domain interactions where the outer domains alternated from being tilted either in a counter clockwise (CCW; conformation 1, blue outer domains, Fig 2B and 2C) or clockwise (CW; conformation 2, pink outer domains, Fig 2B and 2C) manner with respect to the filament axis. Because different subunits on the same protofilament have the same D0/D1 conformation in a supercoiled flagellar filament [28], with each protofilament having slightly different D0/D1 conformations, we compared the outer domain conformational differences by aligning conformations 1 and 2 by domains D0 and D1 (Fig 2D). Between conformations 1 and 2, domains D2 differ from each other by a displacement of about 4–6 Å and domains D3 differed by about 8–15 Å. One protofilament on the outer curve of the filament had all of its outer domains in a single conformation (Fig 2E, gold protofilament). This unique protofilament constitutes a seam or discontinuity in the helical symmetry of the outer domains. This can be seen when comparing the PAO1 flagellar filament power spectrum (dimers with a seam) to that of *C*. *jejuni* which has one flagellin conformation (S4A and S4B Fig). The pattern of outer domain interactions in the PAO1 filament can be quite easily visualized when looking at a helical net (S4C Fig). The seam is generated by the two conformations 1 and 2 alternating along the 5-start helix. Any dimerization of outer domains along an odd-start helix must result in a seam [30].

### A network of inter-domain interactions in *P*. *aeruginosa* PAO1 flagella is required for motility

**Fig 3:**
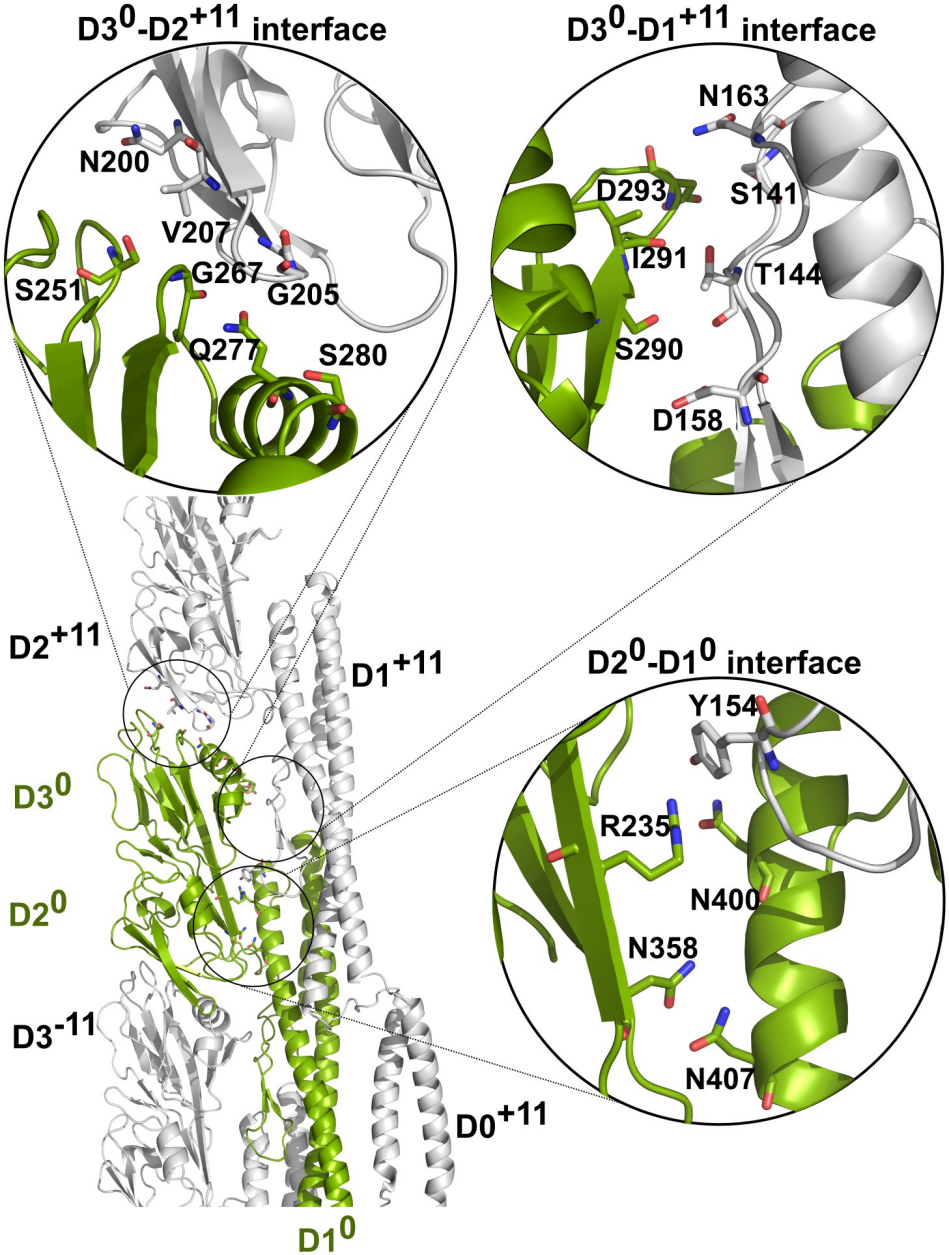
Three interfaces that D2^0^ and D3^0^ form with surrounding domains (highlighted with circles): D3^0^-D2^+11^ (equivalent to D3^-11^-D2^0^), D3^0^-D1^+11^ and D2^0^-D1^0^.

An outer domain in the PAO1 filament forms three interfaces (Fig 3). The first interface results from the dimerization of the neighboring outer domains (e.g., between the D3 domain of one FliC subunit and D2 of the other, D3^0^-D2^+11^ interface) and consist of residues in helix α2, and loop regions 249–253 and 266–269. The second interface is formed between D3 and the β hairpin on the D1 domain of that same neighboring FliC subunit (D3^0^-D1^+11^ interface). Finally, the third interface arises from D2 also forming polar contacts with its own D1 domain (D2^0^-D1^0^ interface).

**Fig 4:**
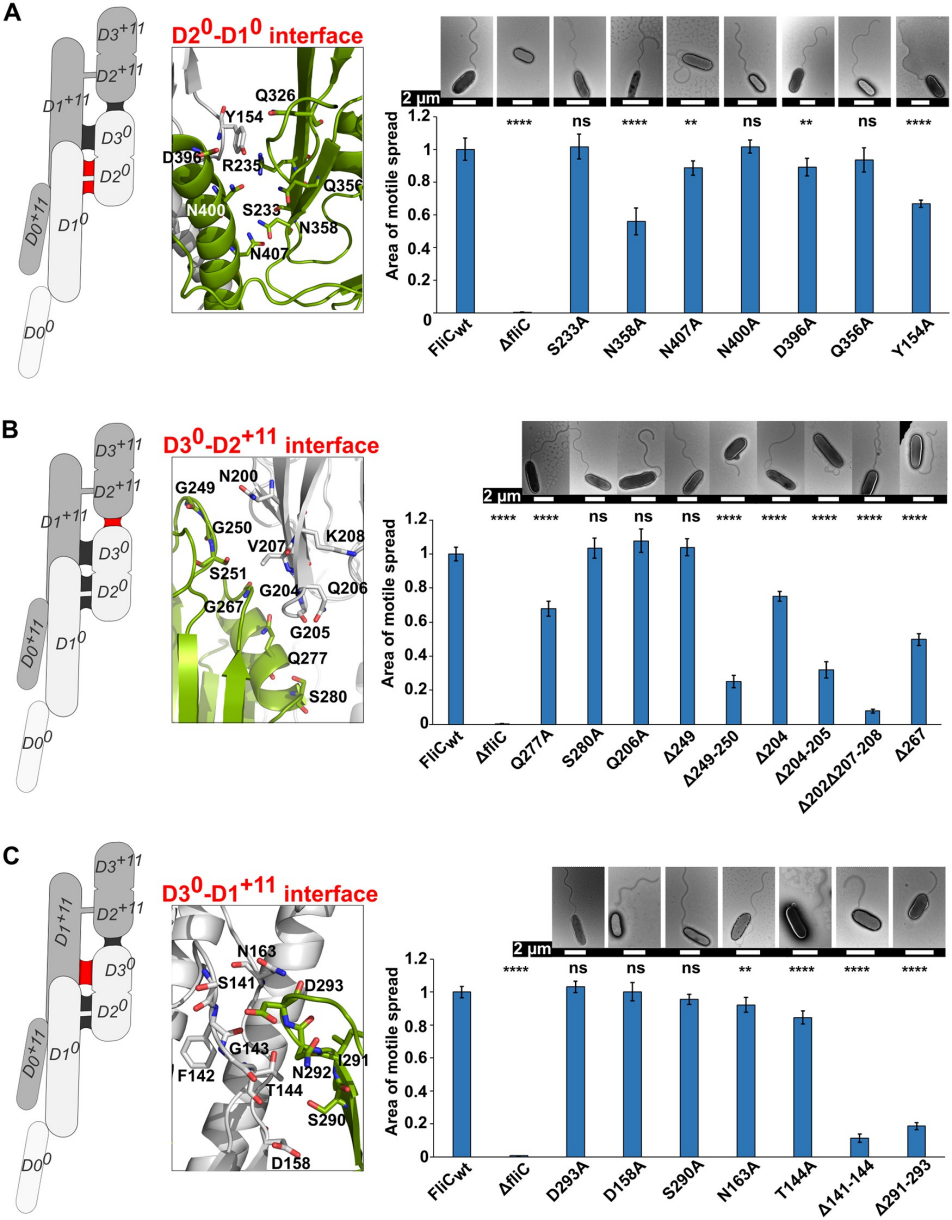
Effects of the FliC mutations on *P*. *aeruginosa* PAO1 swimming motility and filament formation. Swimming motility analysis and negative-stain EM images of PAO1-*ΔfliC* strain complemented with FliC bearing mutations in the D2^0^-D1^0^ interface (A), D3^0^-D2^+11^ interface (B), and D3^0^-D1^+11^ interface (C). Positions of the interfaces are labeled red on the FliC dimer cartoon on the left. Area of motile spread for each strain represents the average of ten replicates and it is normalized to that of the full-length wild type complemented strain (FliC_WT_). Error bars represent standard deviation. Statistical significance was determined by Brown-Forsythe and Welch ANOVA test followed by a Dunnett’s T3 multiple comparison test for data series that passed Shapiro-Wilk normality test, or Kruskal-Wallis test for data series that did not pass normality test (ns—not significant; ** p < 0.01; **** p < 0.0001).

In order to determine the functional importance of the observed D2/D3 interfaces in the filament, we generated a series of *fli*C mutant genes on a plasmid that we used for *P*. *aeruginosa* PAO1-*Δfli*C strain complementation. We then observed the effects of mutations on filament formation and tested swimming motility in an agar-based swimming assay by comparing their motile spread to that of the *P*. *aeruginosa* PAO1-*Δfli*C strain complemented with the wild type gene (Figs 4, S5A–S5R, and S6). We also compared the results of the swimming assays with the swimming speed of the complemented strains in liquid media. For this study, we focused on the polar contacts predicted by PDBePISA (Proteins, Interfaces, Structures and Assemblies) analysis [31] of each of the eleven protofilaments (S7 Fig). FliC mutations were of two kinds—in the case of polar interactions involving side chains we mutated residues to alanine, while in the cases of hydrogen bonds involving main chain atoms, as well as loops that were part of the buried surface areas of the inter-domain interfaces, but did not form hydrogen bonds, we deleted a variable number of residues. Our mutational studies indicated that each one of the inter-domain interfaces within *P*. *aeruginosa* PAO1 filaments—the D2^0^-D1^0^, D3^0^-D2^+11^ and D3^0^-D1^+11^ interfaces—are critical for motility and that, collectively, they form a network of interactions bridging the outer domains to the inner core of the filament that is required for motility, as follows.

In the D2^0^-D1^0^ interface, we mutated seven residues that engage in hydrogen bonds and reside on secondary structural elements in our model into alanine. Although also a part of the D1^0^-D1^+11^ interface, Y154 of FliC^+11^ is predicted to participate in the hydrogen bond network formed by R325, R237, Q399 and N400, which are located in the D2^0^-D1^0^ interface, and we mutated this residue as well. As observed by electron microscopy, all of these mutations resulted in flagellar filament formation (Fig 4A). The majority of these mutations statistically significantly impaired swimming motility compared to wild type. FliC mutants Y154A and N358A exhibited the most substantial decreases in motility, with reductions in motile spread of 35% and 45%, respectively. While the filament length of N358A was equivalent to the wild type, filaments of Y154A were on average longer. Both strains, however, exhibited the same swimming speed in liquid culture as wild type (S3 Table).

In the D3^0^-D2^+11^ interface (Fig 4B), single site alanine mutations had either modest or no effect on motility, however, alanine mutation of Q277 decreased the motile spread by one-third of that of the wild type FliC, while free swimming speed was not affected (S3 Table). Conversely, shortening of the loops by truncation led to significant decreases in motile spread in all but one case. As for the D2^0^-D1^0^ interface, all FliC modifications in the D3^0^-D2^+11^ interface resulted in the formation of flagellar filaments, as observed by electron microscopy. Even in the most extreme case of Δ202Δ207–208, which was nearly entirely immotile in the agar assay, filaments of the wild-type length formed (S3 Table). However, the morphology of the filaments for some mutants differed compared to the wild type. While the filaments in Δ249–250 and Δ204–205 were shorter than the wild type, those of Δ202Δ207–208 were primarily characterized by the loss of an apparent waveform observed for wild type filaments. It should be noted that while Δ204–205 and Δ249–250 on average had shorter filaments, in both cases 25% of the longest filaments had the length that were similar to or larger than the wild-type average of 5.3 μm (S3 Table), suggesting increased fragility of the filaments. The swimming speeds of Δ249–250 and Δ267 were equivalent to the wild type.

While mutations of individual residues in the D3^0^-D1^+11^ interface did not markedly affect PAO1 motility, we observed a profound effect on swimming motility when either of the two loop regions on D3^0^ or D1^+11^ that engage in hydrogen bonds were truncated in order to abrogate these hydrogen bonding networks (Δ293, Δ292–293 and Δ291–293 on D3, and Δ141, Δ141–142, Δ141–143 and Δ141–144 on D1^+11^); again, the formation of filaments was not affected (Figs 4C and S6B). While filaments in Δ141, Δ141–142 and Δ141–143 were comparable to the wild type, filaments of Δ293 and Δ292–293 were longer. Conversely, filaments in Δ141–144 and Δ291–293 were significantly shorter compared to wild type, which could be due to lack of optimal packing in the D3^0^-D1^+11^ interface and a resulting loss of structural integrity of the filament. Similarly to Δ204–205 and Δ249–250, one-fourth of the Δ141–144 cells had filaments around the length of the wild type (4.7–7 μm) (S3 Table).

Regardless of how the above-described mutations affected motility, all resulted in the formation of flagellar filaments, suggesting that each FliC mutant was expressed, exported and properly folded by the bacterium. We confirmed this by Western blot analysis showing that the amount of intracellular FliC and FliC in the culture media was comparable among complemented strains (S8A Fig). The differences in the size of the mutant motile spread were also not due to the difference in their growth rate, since all of them grew at the same rate (S8B Fig). Additionally, we recombinantly expressed and purified all of the full-length (i.e., inclusive of all domains D0 through D3) FliC deletion mutants and tested them for proper folding and thermal stability. As assessed by size exclusion chromatography (S9A Fig), all of them were soluble and monomeric, except for Δ204–205, which was predominantly dimeric. In addition, we subjected each of these deletion mutants to differential scanning fluorimetry (S9B Fig) and determined that their melting temperatures were all similar to that of the wild type. These data indicate that the changes in swimming motility related to mutations or truncations in the complemented *fliC* genes were not a result of substantial FliC protein structural differences, aggregation or instability.

### Type B FliC provides an advantage in swimming through a viscous environment

**Fig 5:**
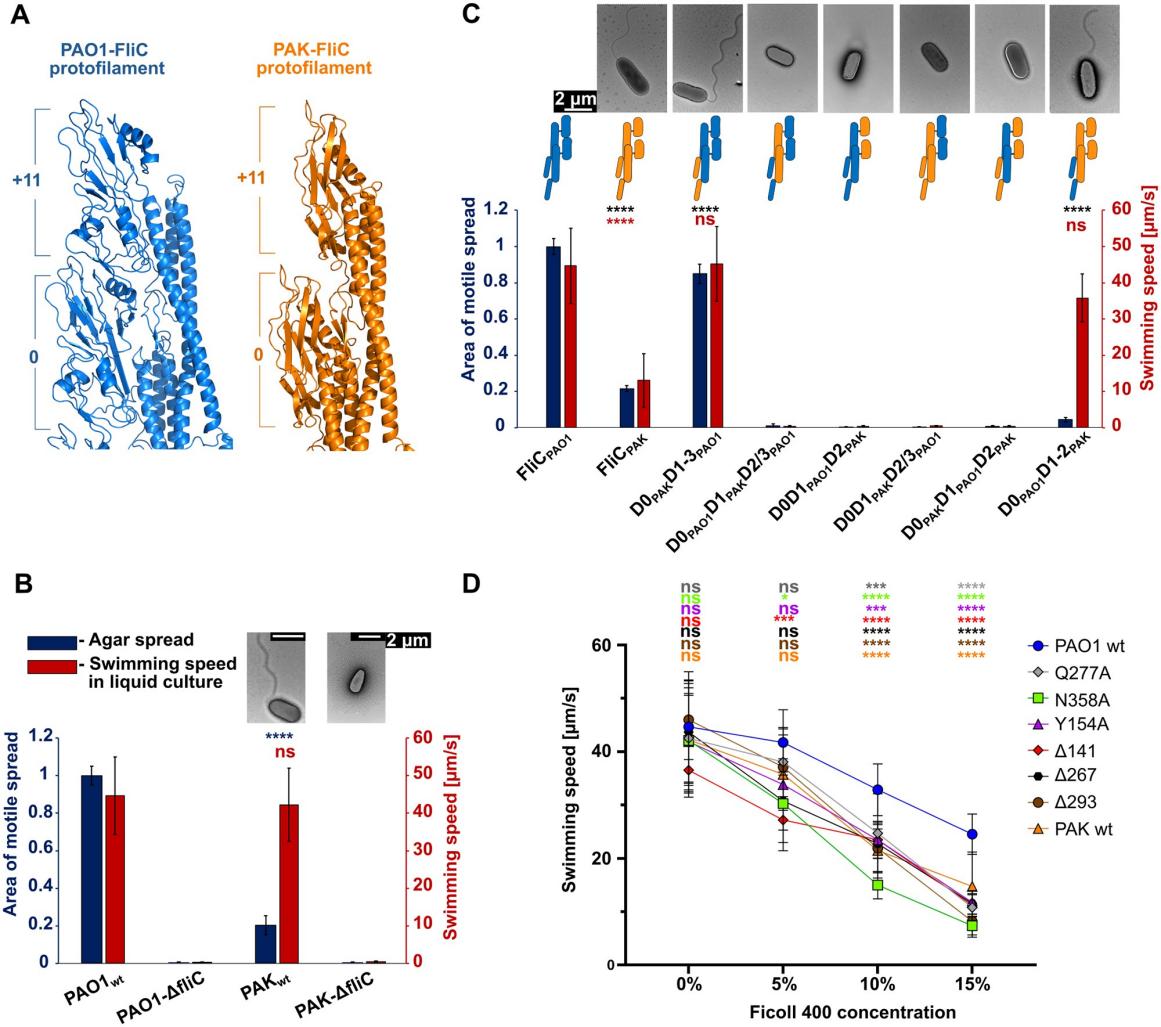
Effects of FliC domain swaps between *P*. *aeruginosa* PAK and PAO1 strains on swimming motility and filament formation. (A) Protofilament comparison of PAO1 (blue) and PAK (orange) strains. Outer domains in PAK are farther apart and do not engage in contacts. (B) Swimming motility and negative-stain EM images of the wild type PAO1 and PAK strains. (C) Swimming motility and negative-stain EM images of PAO1-Δ*fliC* strain complemented with PAK-FliC or FliC in which one or two domains are replaced with equivalent domains of PAK strain. (D) Comparison of average swimming speeds of *P*. *aeruginosa* strains in liquid media complemented with increasing concentration of Ficoll 400. Statistical significance for each condition is in S4 Table Blue bars represents the motile spread on semi-solid agar. Red bars represent average swimming speed in liquid medium (right vertical axis). Area of motile spread for each strain represents the average of ten replicates and it is normalized to that of the PAO1 wild type strain (A), or full-length wild type complemented strain (FliC_WT_) (B). Error bars represent standard deviation. Statistical significance was determined by Brown-Forsythe and Welch ANOVA test followed by a Dunnett’s T3 multiple comparison test for data series that passed Shapiro-Wilk normality test, or Kruskal-Wallis test for data series that did not passed normality test (ns—not significant; * p < 0.05; ** p < 0.01; *** p < 0.001; **** p < 0.0001).

Different strains of *P*. *aeruginosa* contain FliC of either type A or type B and their structures differ significantly. We used the available crystal structure of the type A FliC from the *P*. *aeruginosa* PAK strain to model the PAK filament by superposing D0 and D1 domains of type A FliC to the corresponding domains in our PAO1 filament structure. Our model suggests that the outer domains of the PAK filament in each protofilament are farther apart than those in PAO1 and do not engage in direct subunit-to-subunit interactions (Fig 5A). In order to investigate the consequences of these different filament architectures between the PAO1 and PAK strains, we first compared the motility of PAO1 and PAK wild type strains in liquid and in our agar-based swimming motility assay. Our results showed that while the swimming speeds of the PAK and PAO1 strains in liquid are comparable, the ability of the PAK strain to swim in semi-solid agar was significantly lower than that of PAO1, with a motile spread of only 20% relative to PAO1, even though both strains have fully formed flagella (Figs 5B and S5U).

We next sought to determine whether it was possible to replace type B FliC in PAO1 with type A FliC from PAK. We complemented the PAO1-*ΔfliC* strain with a plasmid-borne type A FliC and tested for motility using the agar-based swimming assay and the presence of filaments by electron microscopy. Our results showed that the PAO1 strain complemented by the type A FliC of PAK, like wild type PAK bacteria, had a motile spread of only 20% compared to the strain complemented by type B FliC of PAO1 and formed substantially shorter flagella (Fig 5C). It is known that species compatibility between FliC and FliD is required for the filament to be formed [17]. Therefore, we wanted to determine the effect of the replacement of the individual domains in PAO1-FliC with the corresponding domains of PAK-FliC. We generated six constructs in which either one or two domains originated from PAK-FliC and the remainder from PAO1-FliC: D0_PAK_D1-3_PAO1_, D0_PAO1_D1_PAK_D2/D3_PAO1_, D0D1_PAO1_D2_PAK_, D0D1_PAK_D2/3_PAO1_, D0_PAK_D1_PAO_1D2_PAK_, D1_PAO1_D2D3_PAK_. Of these six constructs, only the two strains complemented with *fliC* in which both D1 and the outer domains were from the same strain—D0_PAO1_D1D2_PAK_ and D0_PAK_D1-3_PAO1_ –formed filaments (Fig 5C). Replacing the D0 domain of PAO1 with D0 of PAK (D0_PAK_D1-3_PAO1_) led to a decrease in spread of only 15% compared to the knockout strains complemented with wild type *fliC*, comparable to the observed decrease in swimming speed (S3 Table); that is, this complementation resulted in fully restored swimming motility relative to wild type PAO1 (Figs 5C and S5R–S5T). Conversely, replacing D1 and D2/D3 domains of PAO1-FliC with D1 and D2 of PAK (D0_PAO1_D1D2_PAK_) resulted in nearly complete abolition of swimming motility, as indicated by a decrease of motile spread of 95% compared to the wild type. This was in sharp contrast to the almost wild type swimming speed of D0_PAO1_D1D2_PAK_ in liquid (S3 Table). We confirmed by Western blot analysis that all chimeric genes were expressed, and the presence of protein in the media shows that FliC was exported through the flagellar T3SS (S10A Fig), eliminating protein synthesis and secretion as reasons for the absence of filaments in filament-less strains.

Discrepancy between swimming speeds in liquid culture and the agar motile spread of PAO1 and PAK, as well as between PAO1 strains with wild type and mutated FliC, suggested that outer domain interactions could be important for swimming in environments with higher viscosity. Agar matrix is a hydrogel that possesses viscoelastic, rather than viscous, properties arising from polymer network and the aqueous solution [32–34]. Thus, in order to test the effect of viscosity on swimming motility, we recorded the swimming of cells in liquid media with increasing Ficoll 400 concentration using phase-contrast microscopy and determined their swimming speeds. We tested wild type PAO1 and PAK strains, as well as PAO1 Δ*fliC* knockout strains complemented with FliC-Q277A, N358A, Y154A, Δ141, Δ267 and Δ293, strains that exhibited wild-type swimming speeds in the absence of Ficoll 400 but showed significant decrease of motile spread. Our results show that the swimming speed of all tested strains decreased more rapidly with increasing viscosity when compared to the wild type (Fig 5D and S4 Table). In 15% Ficoll 400, the average speed of PAO1 was 25 μm/s, compared to 15 μm/s for PAK. The fastest PAO1-FliC mutant, Δ267, with average speed of 12 μm/s was 50% slower than wild type PAO1, while N358A and Δ293, two mutants with smallest motile spread, had even slower average speeds of 8 μm/s and 7 μm/s, respectively.

These results indicate that the structure of the type B outer domains and their interactions along the protofilament confer a significant motility advantage to the *P*. *aeruginosa* PAO1 strain not only in semi-solid agar, but also in viscous liquid media. Only when the entire network of inter-domain interactions connecting the outer domains to the inner core are present and completely connected in a *P*. *aeruginosa* PAO1 background is the bacterium capable of optimally swimming through the viscous environment.

### Only the C-terminus of *P*. *aeruginosa* PAO1 FliC can be replaced by the equivalent region of *S*. Typhimurium FliC

**Fig 6:**
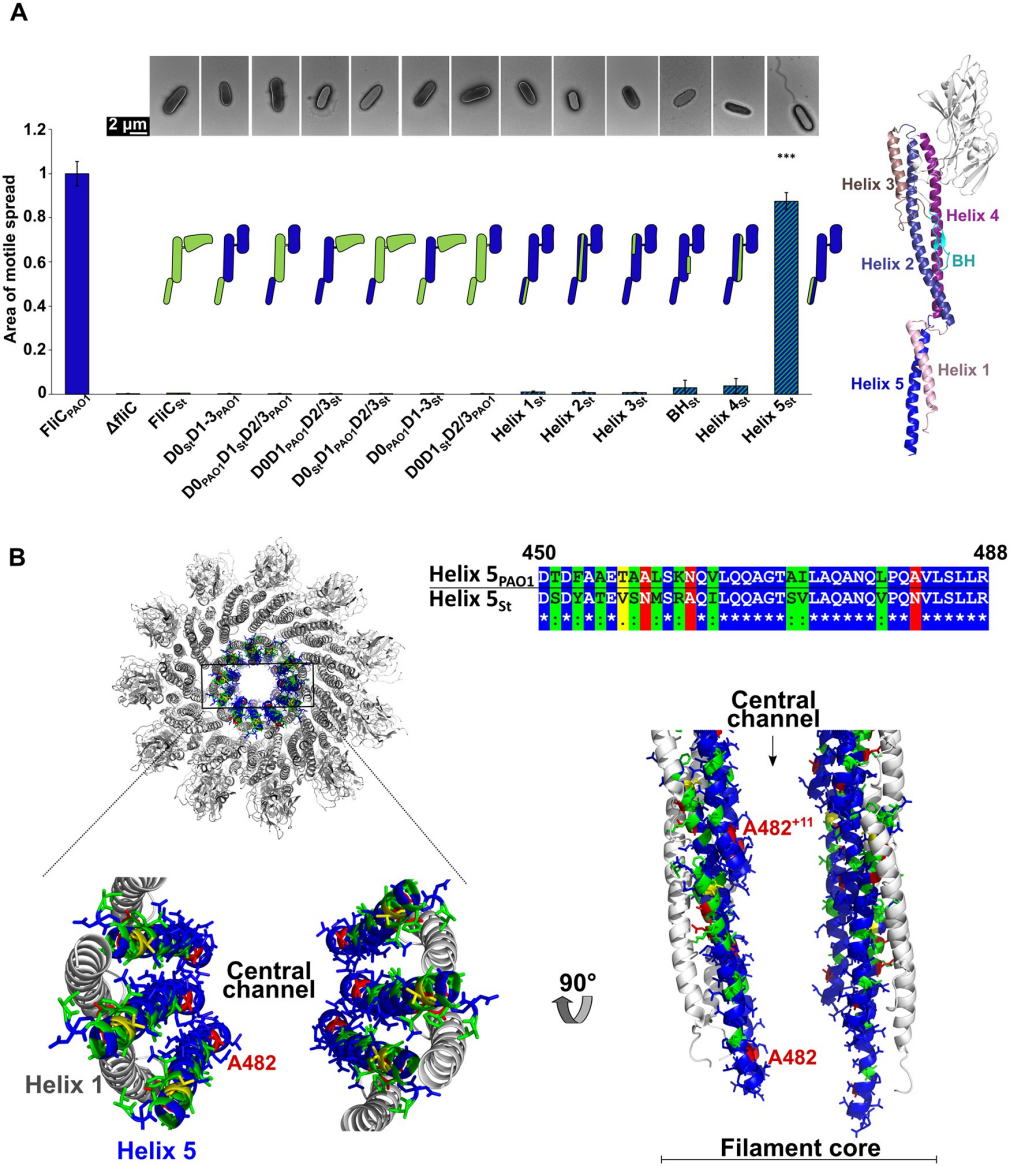
Effects of FliC domain swaps between FliC of *P*. *aeruginosa* PAO1 and *S*. Typhimurium on swimming motility. (A) *Left–*Swimming motility analysis and negative-stain EM images of PAO1-*ΔfliC* strain complemented with FliC in which PAO1 domains (blue) or secondary structural elements are replaced with equivalent regions of *S*. Typhimurium (green). *Right*–Secondary structural elements replaced presented on the structure of PAO1-FliC. BH—beta-hairpin. Area of motile spread for each strain represents the average of ten replicates and it is normalized to that of the full-length wild type complemented strain(FliC_WT_). Error bars represent standard deviation. Statistical significance was determined by Brown-Forsythe and Welch ANOVA test followed by a Dunnett’s T3 multiple comparison test (*** p < 0.001). (B) Cross-section of PAO1 filament showing position of helix 5 in the filament core and sequence alignment of helix 5 from PAO1 and *S*. Typhimurium. Blue—residues that are identical between PAO1 and *S*. Typhimurium. Green—conservative substitutions; yellow and red—non-conservative substitutions.

As previously mentioned, the FliC outer domains in *Salmonella* adopt a different structure than FliC in *P*. *aeruginosa* PAO1 and they do not form contacts with neighboring subunits. Conversely, the FliC inner domains, D0 and D1, exhibit 54% identity and 75% similarity. Therefore, we further expanded our functional screen by generating constructs of FliC chimeras optimized for expression in *P*. *aeruginosa* PAO1 in which we replaced domains D0, D1 and D2/D3 of PAO1-FliC by equivalent domains from *S*. Typhimurium. After complementation of PAO1-Δ*fliC* with these constructs, we observed neither filament formation nor swimming motility (Figs 6A and S5V–S5W). Thus, none of the constructs, including wild type *Salmonella* FliC, could complement for the lack of endogenous FliC in PAO1-Δ*fliC* strain. We next replaced individual helices in D0 or D1, as well as the β-hairpin in D1. Of the resulting six mutants, only the construct in which the C-terminal helix, Helix 5, of PAO1 was replaced by the *Salmonella* sequence exhibited filament formation that were indistinguishable in length and appearance from the wild type filaments (Fig 6A). Moreover, its swimming motility was close to that of wild type. We showed by Western blot analysis that all chimeric FliC proteins were expressed and exported except in the case of full-length Salmonella FliC, as well as chimeras with D0 or Helix 1 from Salmonella, confirming the role of N-terminus as a signal sequence for flagellin secretion (S10B Fig).

The C-terminal helix forms part of the inner core of the filament, lining the central channel, making it the only structural part of the filament in contact with the incoming FliC molecule that is being transported to the filament tip (Fig 6B). Of the 40 residues that comprise this helix, 14 differ between PAO1 and *S*. Typhimurium, four of which are non-conservative substitutions. Only the non-conservative partially buried A482 residue (N497 in *Salmonella*) is exposed to the channel lumen. The other three non-conservative substitutions, as well as the majority of conservative substitutions are located on the side of Helix 5 forming contacts with other inner core helices. This suggests that while changes in lateral contacts of the inner core are tolerable, the specific sequence lining the lumen could be important for transport of FliC through the channel.

### The ridged flagellar filaments characteristic of *P*. *aeruginosa* PAO1 are found in diverse bacteria

**Fig 7:**
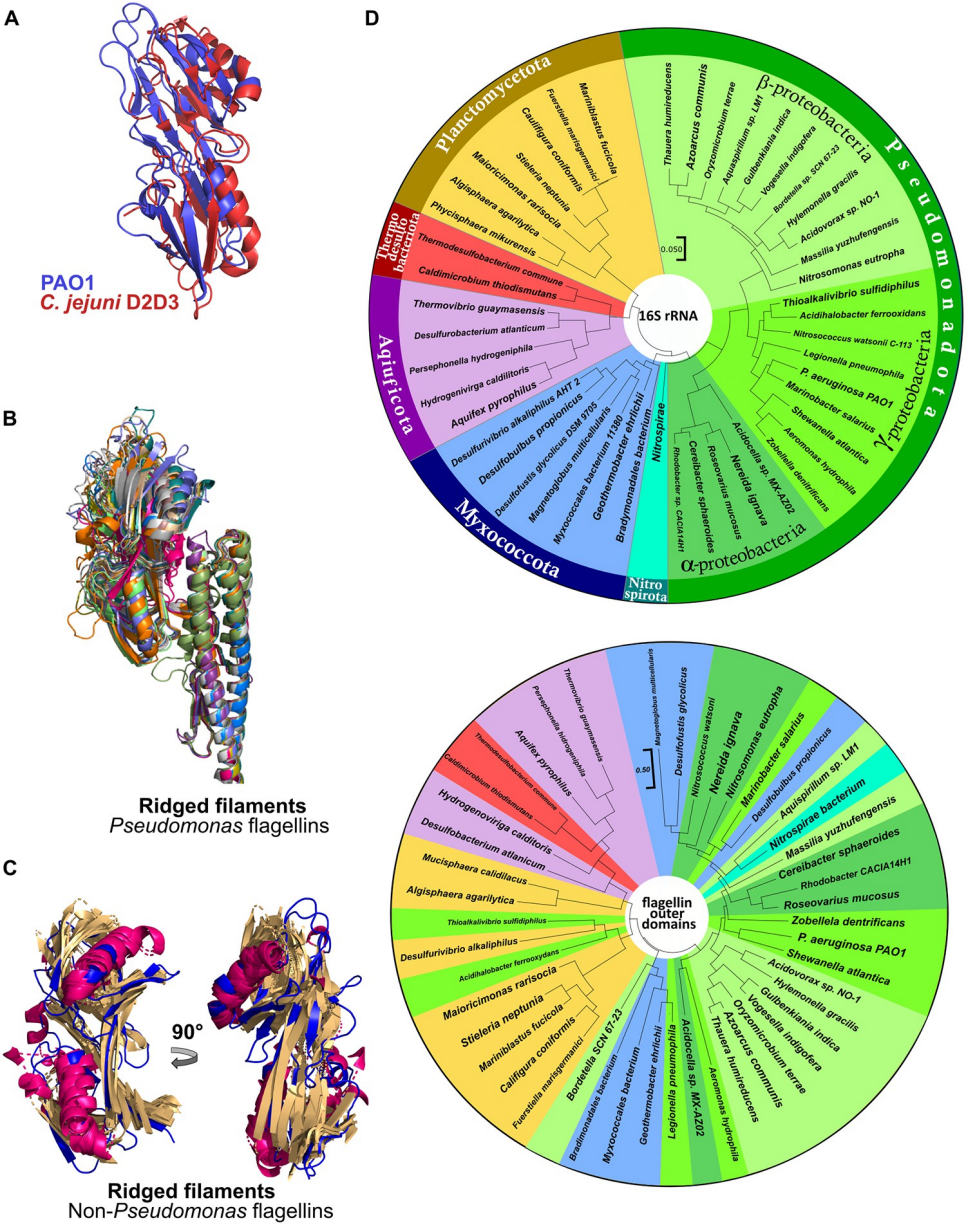
PAO1-like flagellin fold predicted by AlphaFold in different bacterial species. (A) Superposition of D2 and D3 domains of *C*. *jejuni* flagellin FlaA and PAO1-FliC. (B) Superposition of FliC molecules of *Pseudomonas* species other than *P*. *aeruginosa*. (C) Superpositions of flagellin outer domains of non-*Pseudomonas* species. Unstructured loop regions were removed for clarity. (D) Phylogenetic trees based on 16S rRNA (top) and flagellin outer-domain protein sequences (bottom). Species are grouped into bacterial phyla according to the currently accepted ICNP nomenclature.

The structure of the flagellar filament of *P*. *aeruginosa* PAO1 is markedly different from that of *S*. Typhimurium. In *Salmonella*, outer domains D2 and D3 are clearly separated from each other and from neighboring outer domains and do not interact [19]. Conversely, the recently published structure of the *C*. *jejuni* filament shows a flagellin with three distinct outer domains, with domains D2 and D3 having similar topology to that of D2 and D3 of PAO1 (Fig 7A) [23]. The outer domains of *C*. *jejuni* filament also engage in interactions through D2^0^-D3^+11^ interfaces forming the ridged filament that we observe in PAO1. Recently, AlphaFold structure predictions for over 200 million proteins became available [35]. Thus, in order to explore the variety of outer domain folds and identify other flagellins with a fold similar to PAO1-FliC, we turned to the newly available database of AlphaFold-predicted structures available through Uniprot. We searched for flagellin sequences in Uniprot and visually inspected the AlphaFold-predicted structures of those with a size ranging from 450 to 550 amino acid residues.

First, we confirmed that AlphaFold [36,37] was able to properly predict our crystal structure of PAO1 D2/D3, which it did with an RMSD of 0.6 Å, suggesting that AlphaFold can predict PAO1-like flagellins with high confidence. We then searched for flagellin structures from *Pseudomonas* species other than *P*. *aeruginosa* and found that at least 10 of these have an outer domain that corresponds to type B FliC (Figs 7B and S11). Next, we expanded our search for flagellin sequences from the entire bacterial kingdom, inclusive of all 42 validated bacterial phyla [38]. Among these bacterial phyla, we found flagellin sequences for 31 of them, the remaining 11 phyla being presumed non-flagellated bacteria. Of the flagellated bacteria, we identified at least 46 species in 6 phyla in which flagellins have a PAO1-like outer domains, including Aquificota, Myxococcotta, Pseudomonadota, Planctomycetota and Thermodesulfobacteriota (Fig 7C and 7D). Phylum Pseudomonadota (Proteobacteria) had the largest number of PAO1-like flagellins, with species belonging to the Alpha-, Beta-, and Gamma-proteobacteria classes. We only identified these PAO1-FliC-like proteins by visual inspection of AlphaFold-predicted structures of FliC proteins of similar length, as these outer domains do not exhibit any significant similarity on the amino acid sequence level (S12 Fig). These results suggest that PAO1-like flagellins that form ridged filament are widespread among bacteria. That flagellins of genetically distant bacteria evolved to retain the same fold of their outer domains despite lacking any measurable sequence similarity suggests that the ridged filament architecture confers advantages to the bacteria that express them, such as for swimming in viscous environments.

## Discussion

Bacterial flagella are highly complex and dynamic molecular machines that translate energy generated by a proton motive force or a sodium gradient into rotation and, ultimately, thrust in order to propel bacteria through liquid environments. The great majority of each flagellum is composed of many copies of the flagellin protein, in many bacteria called FliC, that form the flagellar filament. The function of the filament is manyfold: primarily, by forming a macroscopic supercoil or helical propeller the filament generates thrust by means of an Archimedean screw mechanism; secondarily, it mediates adhesion to host surfaces and other bacteria to promote colonization and biofilm formation, respectively, and may be involved in a variety of other functions, such as immune escape. Flagellar filaments can be neatly divided structurally into the two highly conserved inner domains, D0 and D1, which form the inner core of the filament, and a diverse number of outer domains, which decorate the inner core.

The role of the inner domains is increasingly well understood. They create a channel through which flagellin subunits exported through the flagellar type 3 secretion system (T3SS) travel on their way to encountering the FliD filament cap, folding and thereby extending the flagellar filament from its distal end. They are also required for the supercoiling that generates thrust in what would otherwise simply be a spinning rod. Since growing a filament that subsequently generates thrust is the minimum requirement for swimming motility, it should come as no surprise that some motile bacteria, such as *B*. *subtilis*, have evolved to express flagellins containing only D0 and D1 inner domains. Also unsurprising is that these inner domains are highly conserved, in sequence and in structure, throughout flagellated bacteria.

Most flagellated bacteria, however, express flagellins with one or more additional outer domains. Moreover, these outer domains are highly divergent in sequence and, as an increasing number of structural studies have demonstrated, in domain conformation and overall supramolecular architecture. While these remain difficult to fully categorize, some of the observed architectures include splayed, as in *S*. Typhimurium [19,20], ridged, as in *P*. *aeruginosa* PAO1 [25], screw-like, as *Sinorhizobium meliloti* (24), and sheathed, as in enterohemorrhagic *E*. *coli* O157:H7 (EHEC) [24]. While it has been known for decades that flagellin outer domains are involved in adhesion and biofilm formation, their potential role in motility is only recently becoming appreciated. Indeed, a recent study [24] has implicated a role for the dimeric interface between distal D4 domains of the EHEC filament in motility, suggesting that bacteria that inhabit viscous environments are conferred a motility advantage on account of their interconnected outer domains.

Here we show that FliC outer domain interactions: (1) can involve more extensive networked interactions than just intermolecular contacts in outer domain interfaces; (2) provide a motility advantage to bacteria in viscous versus fluid environments; and (3) are more widespread throughout the bacterial kingdom than previously appreciated. Using *P*. *aeruginosa* PAO1 as a model system of ridged flagellar filaments, we dissected interactions involving its D2 and D3 outer domains and their effects on motility. As a consequence of the architecture of these ridged filaments, these outer domains create a network of interfaces along the protofilament from the inner core, through the outer domains and back to the inner core. By measuring motility of *P*. *aeruginosa* PAO1 mutant filaments, we found that each one these interfaces—between D1^0^ and D2^0^, connecting the inner core to the outer domains; between D3^0^ and D2^+11^, bridging the outer domains along the protofilament; and between D3^0^ and D2^+11^, reconnecting the outer domains to the inner core—is required for motility. Together, these interfaces create a network of interactions upon which motility depends.

To form this unique outer domain network the flagellin proteins must adopt three slightly different conformational states. This contrasts starkly with the outer domain dimerization described previously in bacterial flagellins where the outer domain conformations involve a 180° rotation of the domains creating a dimer interface with D1 symmetry [24]. The conformational states adopted by the PAO1 B type flagellin result in the formation of a rigid flagellar filament structure with a seam on the outer curve of its supercoiled structure.

To demonstrate that FliC outer domains conferred a motility advantage to bacteria in viscous environments, we complemented *P*. *aeruginosa* PAO1 with PAO1/PAK chimeric filaments. Unlike the ridged filaments in *P*. *aeruginosa* PAO1, *P*. *aeruginosa* PAK exhibits splayed filaments in which the outer domains do not make contacts with one another along the protofilament. When we measured motility in liquid, wild type PAO1 and PAK, as well as all chimeras that produced flagella, were similarly motile. Conversely, when challenged with viscous liquid media or a gel-like soft agar environment, wild type PAO1 was substantially more motile than wild type PAK. Of those chimeras that produced flagella, only that which included the D1, D2 and D3 domains from PAO1 –thereby reconstituting the entire network of interactions between the inner core and outer domains in wild type PAO1 –was similarly motile to wild type PAO1.

We found that ridged flagellar filaments, and consequently the networked interactions between the inner core and outer domains, are not unique to *P*. *aeruginosa* PAO1. Our computational predictions show that not only are ridged filaments found extensively throughout *Pseudomonas* species, at least 46 species outside of the *Pseudomonas* genus have similarly folded outer domains. While sequence alignment reveals no significant identity between these outer domains, the structures of these outer domains have been conserved. Due to the laborious method by which we identified these similarly-folded FliC proteins, which relied on visual inspection of individual AlphaFold-predicted structures, this undoubtedly represents an undercount; ridged flagellar filaments may be even more widespread throughout bacteria than we have found here. It is also reasonable to believe that the total number of different outer domain structures is smaller than suggested by sequence diversity of outer domains and that filaments could be classified into just a handful of architectures, minimally including: naked (e.g., *B*. *subtilis*), splayed (e.g., *S*. Typhimurium), ridged (e.g., *P*. *aeruginosa* PAO1), screw-like (e.g., *S*. *meliloti*) and sheathed (e.g., EHEC) filaments. Different architectures could reflect the specific needs of distinct groups of bacterial species.

The explanation for the existence of ridged filaments in PAO1 and *C*. *jejuni*, and consequently in other species, could be in the functional properties of the flagellum. The flagellar motor causes rotation of the filament, creating thrust and enabling bacteria to swim forward. The motor consists of two distinct molecular assemblies, the membrane-embedded rotor, and a torque-generating stator. Stators are composed of a variable number of stator units, with each unit being a complex of the proteins MotA and MotB. In *Salmonella*, the stator consists of 11 subunits. Conversely, the *C*. *jejuni* motor has up to 17 stator units [39]. This translates to a much higher swimming speed close to 100 μm/s, compared to 25 μm/s of *Salmonella*. *C*. *jejuni* also swims faster in moderately viscous fluid than in low viscosities [40]. While not much is known about the number of stator units in *P*. *aeruginosa*, a recent cryo-electron tomography analysis showed that its motor is wider than the motor of *S*. Typhimurium and includes additional prominent densities adjacent to the P- and L-rings made of the MotY homolog [41], a protein that is a part of the *Vibrio alginolyticus* Na^+^-driven motor. Additionally, *P*. *aeruginosa* has two types of stator units, MotA/MotB for swimming in low viscosity, and MotC/MotD for swimming in higher viscosity [42]. All these specificities of the *P*. *aeruginosa* motor would result in generation of greater maximum torque, allowing bacteria to swim at higher velocities through more viscous media [41]. Intriguingly, the PAO1 outer domains assemble into a structure with a seam while the *C*. *jejuni* outer domains do not. In *C*. *jejuni* the singular outer domain conformation along the 11-start protofilaments might allow for greater stability due to their weakened protofilament interface in domain D1 [23]. The results in this study clearly show that the outer domains of PAO1 flagellar filament play a role in motility by allowing the bacterium to navigate a viscous environment in a more robust manner compared to the PAK flagellar filament.

Although the maximum swimming speed of *P*. *aeruginosa* of 50 μm/s is half the speed of *C*. *jejuni*, it is still twice that of *S*. Typhimurium [43]. Both *P*. *aeruginosa* and *C*. *jejuni* have polar flagella. Due to the higher force and load to which their filament is subjected, it is reasonable to expect a much tighter packing to preserve its structural integrity. As such, outer domains with extensive inter-domain interactions could stabilize the filament while swimming in the viscous environment. The ridged outer domains might also provide a stability to PAO1 and *C*. *jejuni* flagellar filaments, as both species wrap their flagella around their cell body and appear to undergo limited polymorphic transitions [44,45]. The lack of connectedness of the PAK outer domains together with its poor relative motile spread in semi-solid agar suggests that its filament architecture is poorly adapted to swimming in a viscous environment with its polar flagellum. Unlike monotrichously flagellated *Pseudomonas* species, *S*. Typhimurium is peritrichously flagellated, and its individual filaments bundle during straight forward swimming. A looser outer domain structure could serve as a mechanism for easier bundling and unbundling when the change of direction is needed.

## Materials and methods

### D2-D3 purification and crystallization

The sequence of *fliC* from *P*. *aeruginosa* PAO1 between residues 178 and 395 containing D2 and D3 domains (FliC_D2D3_) was cloned into a pGEX-5x-2 plasmid with the GST tag on N-terminus followed by a TEV protease recognition site. The plasmid was introduced into BL21(DE3) cells. For the native protein expression, cells were grown in LB medium and the expression was induced with 1 mM IPTG, after which the cells were grown for another 3 hours at 37°C. Selenomethionine-labeled (SeMet) FliC_D2D3_ was expressed for 6 hours after the induction at 37°C in M9 medium containing the amino acids lysine (100 mg/L), phenylalanine (100 mg/L), threonine (100 mg/L), isoleucine (50 mg/L) and valine (50 mg/L) to suppress methionine synthesis, and 60 mg/L of selenomethionine. The cells were harvested, resuspended in PBS and sonicated. The soluble fraction of the lysate was applied to the Glutathione Sepharose beads (GE Healthcare), washed and eluted with PBS containing 25 mM of reduced glutathione. GST tag was removed by overnight incubation with TEV protease followed by affinity chromatography using Ni-NTA beads to remove TEV protease, and size-exclusion chromatography (Superdex 200, GE Healthcare). SeMet-FliC_D2D3_ crystallized in 1.9 M ammonium sulfate and 0.1 M sodium Citrate pH 5.5, while native FliC_D2D3_ crystallized in 2 M ammonium sulfate and 0.1 M Tris pH 7.5.

### D2-D3 crystallization and structure determination

Data sets were collected on the BL12-2 beamline of the Stanford Synchrotron Radiation Lightsource (SSRL) equipped with the Pilatus 6M detector (Dectris). For the selenomethionine-labeled FliC, we collected 3 data sets at peak (λ = 0.97929 Å), high-energy remote (λ = 0.9116 Å) and inflection (λ = 0.9795 Å) wavelengths. Data were indexed, integrated and scaled using the XDS program package [46]. The MAD phasing was performed using SHELX program in CCP4i package [47] and the initial model was built with Buccaneer [48]. The native dataset was collected at λ = 0.97946 Å. Data were phased by molecular replacement with MOLREP [49], using the previously obtained model from the anomalous scattering. The final model at 1.47 Å resolution was refined in several rounds using REFMAC5 [50] and manual editing in Coot [51], and has been deposited to the PDB with the entry code 8ERM.

### Cryo-EM reconstruction of native PAO1 filament

*P*. *aeruginosa* PAO1 was grown in LB medium at 37^0^ C until OD_600_ = 1.5, after which cells were harvested and resuspend in 30 mL of cold PBS. Filaments were sheared off using a Waring commercial blender for 1 minute, followed by centrifugation at 12000x*g* for 10 minutes. Supernatant was collected and concentrated to the final volume of 1 mL. For EM grid preparation, 3.5 μL aliquots of flagellar filaments were applied to Lacey carbon grids and were subsequently plunge frozen using a Vitrobot Mark IV plunge freezers. The blotting time was 3 seconds and the blot forces ranged from 3–6. Imaging was done on a Fisher Scientific Glacios equipped with a Falcon IV direct electron detector with a pixel size of 0.92 Å and a total dose per movie of 50 e^-^/Å^2^. Images were then processed using cryoSPARC [52]. Initial reconstruction was done imposing helical symmetry. Reconstruction of the flagellar filament without the imposition of helical symmetry was then performed as described previously [24,28]. Initial attempts to reconstruct the PAO1 filament with cryoSPARC’s Helical Refinement program using a featureless cylinder were unsuccessful. A ~3.8 Å helical map of the PAO1 filament was obtained after using a starting deposited map of domains D0 and D1 of another flagellar filament (EMD-27064) low-pass filtered to a modest resolution. This helical map was then used as input for the cryoSPARC 3D variability program. The 3D variability results were displayed as clusters and the best cluster showed a clear curvature with multiple outer domain conformational states. The particles and map from this cluster were then subject to local refinement followed by CTF refinement and local refinement again. The final volume had a “Gold-standard” 0.143 map:map Fourier shell correlation (FSC) of 4.2 Å from 29,945 particles and a 0.5 map:model FSC of 5 Å.

### Full filament model refinement

A composite model of the PAO1 flagellin was fit into the cryo-EM density map, and the outer domains where adjusted to fit the density using UCSF Chimera [53] and Coot [54]. After flagellin subunits were roughly fit into the map, the filament model was refined against the map using Phenix Real Space Refinement [55]. The map and model are available as EMD-40765 and PDB 8SUG.

### *ΔfliC*-PAO1 complementation

Wild-type and *ΔfliC* strains *P*. *aeruginosa* PAO1 were obtained from the Manoil Lab at the University of Washington, while *P*. *aeruginosa* PAK strains were kindly provided by Joanna Goldberg from Emory University. Different constructs of *fliC* genes were cloned into the pBBR1 plasmid, which was then transformed into the knockout strains by electroporation [56].

### Swimming assays

Swimming motility assays of *Pseudomonas aeruginosa* strains were performed as described in [57]. Plates were incubated at 37 ^0^C for approximately 20 hours. The area of each bacterial swim circle was quantified using the software ImageJ [58]. Ten replicates were performed for each complemented strain, and the average and standard deviations were determined. Statistical significance was determined by Brown-Forsythe and Welch ANOVA test followed by a Dunnett’s T3 multiple comparison test using GraphPad Prism version 9.2.0 for Windows (GraphPad Software, San Diego, California USA, www.graphpad.com).

### EM analysis

Bacteria were grown in Luria Broth liquid culture overnight at 37°C and immobilized on a Formvar grid (Electron Microscopy Sciences) for 1 min 30 sec and the samples were negatively stained with 0.5% (w/v) Phosphotungstic acid (PTA) and imaged using a FEI Talos 120 KV electron microscope. Filament lengths were calculated using ImageJ program [58], and the median of 30 different measurements was reported. Raw images are available at the Emory Dataverse open data repository (https://doi.org/10.15139/S3/FXGXYY).

### Swimming velocity determination

Cells from overnight culture were diluted into the LB medium to an OD600 of 0.05 and incubated for 30 minutes at 37°C. For swimming in viscous media, cells were diluted into the LB medium supplemented with 5, 10 or 15% Ficoll 400. Cells were observed and recorded in a phase-contrast mode on Lionhear FX automated microscope (Biotek) using a wet mount technique. The recordings were analyzed using the manual tracking plugin in the image processing package Fiji [59]. Average speed for each strain was determined using 30 fastest tracks. Image sequences are available at the Emory Dataverse open data repository (https://doi.org/10.15139/S3/KGXDRA).

### Protein purification and differential scanning fluorimetry (DSF)

Genes of the wild type FliC and deletion mutants were subcloned into pET28-a vector with C-terminal polyhistidine tag. Plasmids were transformed into *E*. *coli* BL21(DE3) cells, grown in LB medium at 37^0^ C until OD_600_ = 0.6. After induction with 1 mM IPTG, cells were grown for another 4 hours at 37^0^ C. Clarified lysate was applied to NiNTA resin (Qiagen) and eluted with 500 mM imidazole in PBS buffer, followed by size-exclusion chromatography using Superdex 200 Increase 10/300 GL column (Cytiva). For DSF analysis, 5 μM of protein in PBS was mixed with SYPRO Orange dye (ThermoFisher) to a final concentration of 25X. The experiment was performed on a real-time thermal cycler for a temperature range between 25^0^ and 90^0^ C.

### Western blot

Cells were grown in LB medium at 37^0^ C until reaching final OD_600_ = 1. For each mutant 1 mL samples were taken, cells harvested at 4000x*g* for 5 minutes. Leftover media was precipitated using acetone and resuspended in 100 μL of PBS. After SDS-PAGE, proteins were transferred on a nitrocellulose membrane using iBlot 2 (Invitrogen). Membrane was blocked using 5% milk, incubated with PAO1 or PAK FliC antisera [60] (kind gift from Dr. Joanna Goldberg), followed by incubation with PE-labeled secondary goat anti-Rabbit antibody (P-2771MP, ThermoFisher). Membrane with samples D0_PAO1_D1-3_St_, D0_St_D1_PAO1_D2/3_St_ and D0D1_St_D2/3_PAO1_ was incubated with primary *S*. Typhimurium flagellin FliC Detection antibody (clone X5A12) (InvivoGen), followed by HRP-conjugate secondary anti-Mouse antibody.—Membranes were visualized using ChemiDoc MP (Bio-Rad).

## Supporting information

S1 Fig Structure of the outer domains of *P*. *aeruginosa* PAO1 FliC. (A) Stereo image of a portion of the 2Fo—Fc electron density map of native FliC_D2D3_ 2Fo—Fc electron density map. (B) Comparison of D2 and D3 domains of *P*. *aeruginosa* PAO1 and *S*. Typhimurium. (C) Topology map of FliC_D2D3_. blue—domain D2; red—domain D3; yellow–β-strand shared between D2 and D3 domains.
(PNG)

S2 Fig Cryo-EM workflow for structural determination of the PAO1 flagellar filament. The general workflow for structural determination performed in cryoSPARC is shown here. It is important to note that the helical reconstruction was rather unusual in that an 8 Å starting D0/D1 map was required for a good final density map. For more detailed information please consult the methods section.
(PNG)

S3 Fig A. Local resolution estimate for the PAO1 flagellar filament. Left image shows an axial view of the filament. The middle image shows the surface of the filament. The right image shows the core of the filament. B. Fourier shell correlation (FSC) curves for the PAO1 filament reconstruction. FSC used in the curves is the gold standard 0.143 map:map FSC (GFSC).
(PNG)

S4 Fig Power spectra and geometry of the *P*. *aeruginosa* PAO1 filament. (A) Power spectrum of a standard monomeric bacterial flagellar filament from *Campylobacter jejuni*. (B) Power spectrum of the PAO1 flagellar filament. As shown previously [25], the number of layer lines present are double that of a typical flagellar filament, indicative of a dimerization of subunits with an asymmetric unit containing a dimer. (**C)** Helical net showing the arrangement of the different PAO1 flagellin conformations along the filament assuming a perfectly straight flagellum with helical symmetry for domains D0 and D1. Each dot represents a subunit and the colors correspond to the conformations shown in Fig 2. The pink and blue dashed line represents a single 5-start helix along which is broken by the seam (gold line) along the outer domains.
(PNG)

S5 Fig Agar plates showing motile spread of *P*. *aeruginosa* PAO1-Δ*fliC* strain complemented with different FliC mutants. (PNG)

S6 Fig Motile spread of all mutants tested. (A) Alanine mutants. (B) Deletion mutants. Negative-staining EM images presented are the images of mutants not included in Fig 3.
(PNG)

S7 Fig Sum of polar bonds between FliC^0^ and FliC^+11^ from all protofilaments identified by PISA server. Different shades of green and gray represent different domains and corresponding residues.
(PNG)

S8 Fig Expression and export of FliC in mutant strains. (A) Anti-FliC western blot showing the presence of FliC in cells and in the media. (+) positive control, purified recombinant FliC from P. aeruginosa PAO1. (B) Growth curves for the subset of tested mutants.
(PNG)

S9 Fig Folding and thermal stability of recombinant FliC mutant proteins. (A) Size-exclusion chromatogram for recombinantly purified wild type FliC and 7 deletion mutants. (B) Fluorescence emission and the first derivative obtained from differential scanning fluorimetry (DSF) experiment.
(PNG)

S10 Fig Anti-FliC western blot showing the presence of FliC in cells and in the media. (A) PAO1-PAK FliC chimeras. (B) PAO1-S. Typhimurium chimeras. (C)–cells; M—media; St–*Salmonella* Typhimurium. Expected FliC band in red square. Variation in sizes is due to different molecular weight of three flagellins: PAK-FliC– 40 kDa, PAO1-FliC– 49 kDa, St-FliC– 52 kDa.
(PNG)

S11 Fig Protein sequence alignment of outer domains of P. aeruginosa PAO1and 10 other species of the genus *Pseudomonas* with PAO1-like flagellin. (PNG)

S12 Fig Protein sequence alignment of outer domains of *P*. *aeruginosa* PAO1 and 46 species with PAO1-like flagellin. (PDF)

S1 Table Data collection, phasing and refinement statistics for MAD (SeMet) structures (PDF)

S2 Table Refinement statistics for cryo-EM map and model of *P*. *aeruginosa* PAO1 filament (PDF)

S3 Table Filament length, swimming speed and motile spread of different *P*. *aeruginosa* strains. (PDF)

S4 Table Swimming speed of different *P*. *aeruginosa* strains in increasing concentration of Ficoll 400. (PDF)
