# Supplementary figures and images for: An unbroken network of interactions connecting flagellin domains is required for motility in viscous environments
Source: PLoS Pathog. 2023 May 30;19(5):e1010979. doi: 10.1371/journal.ppat.1010979 (PMC10256154; doi:10.1371/journal.ppat.1010979)

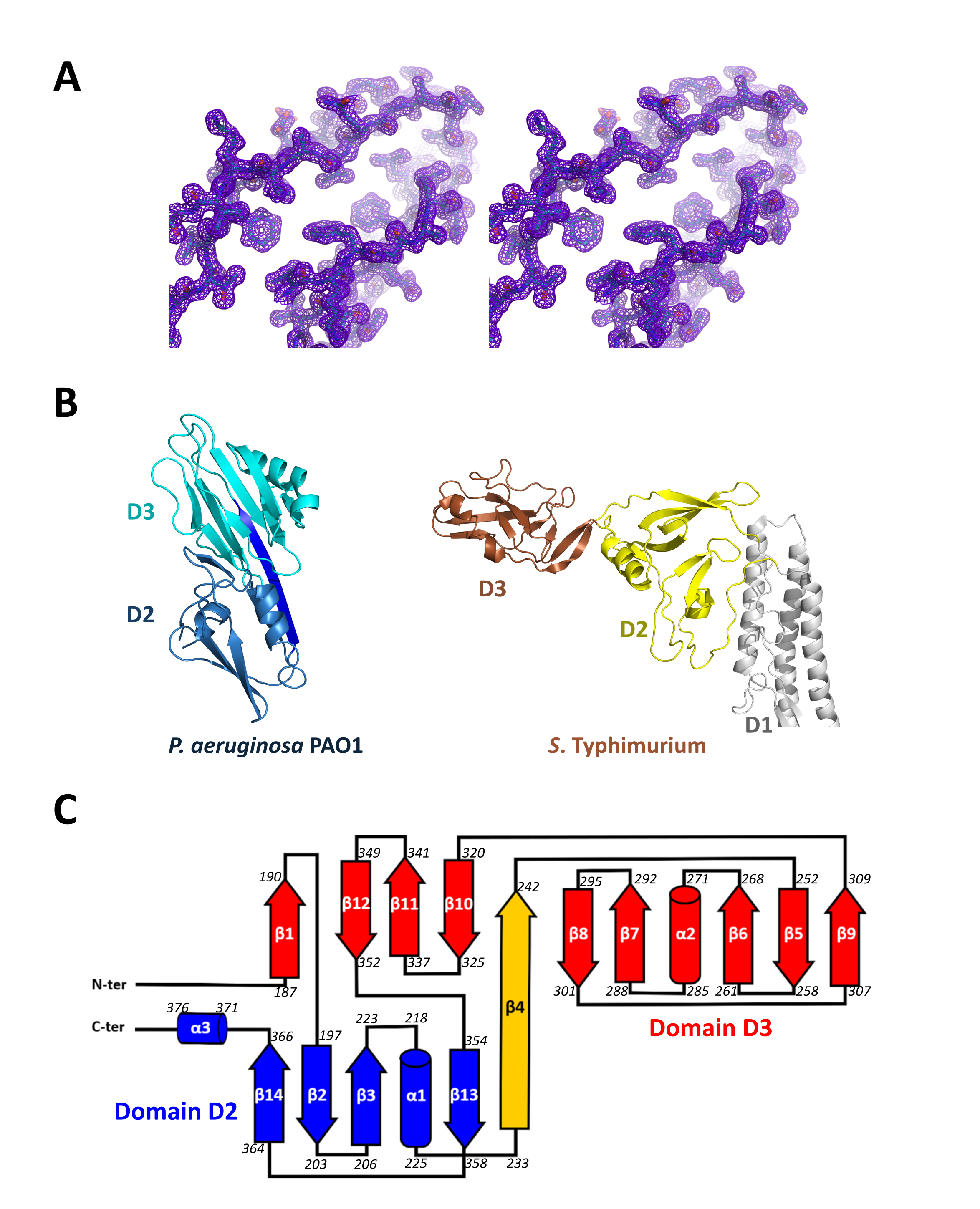

Supplement: S1 Fig — (A) Stereo image of a portion of the 2Fo—Fc electron density map of native FliCD2D3 2Fo—Fc electron density map. (B) Comparison of D2 and D3 domains of P. aeruginosa PAO1 and S. Typhimurium. (C) Topology map of FliCD2D3. blue—domain D2; red—domain D3; yellow–β-strand shared between D2 and D3 domains. (PNG) [file ppat.1010979.s001.png]

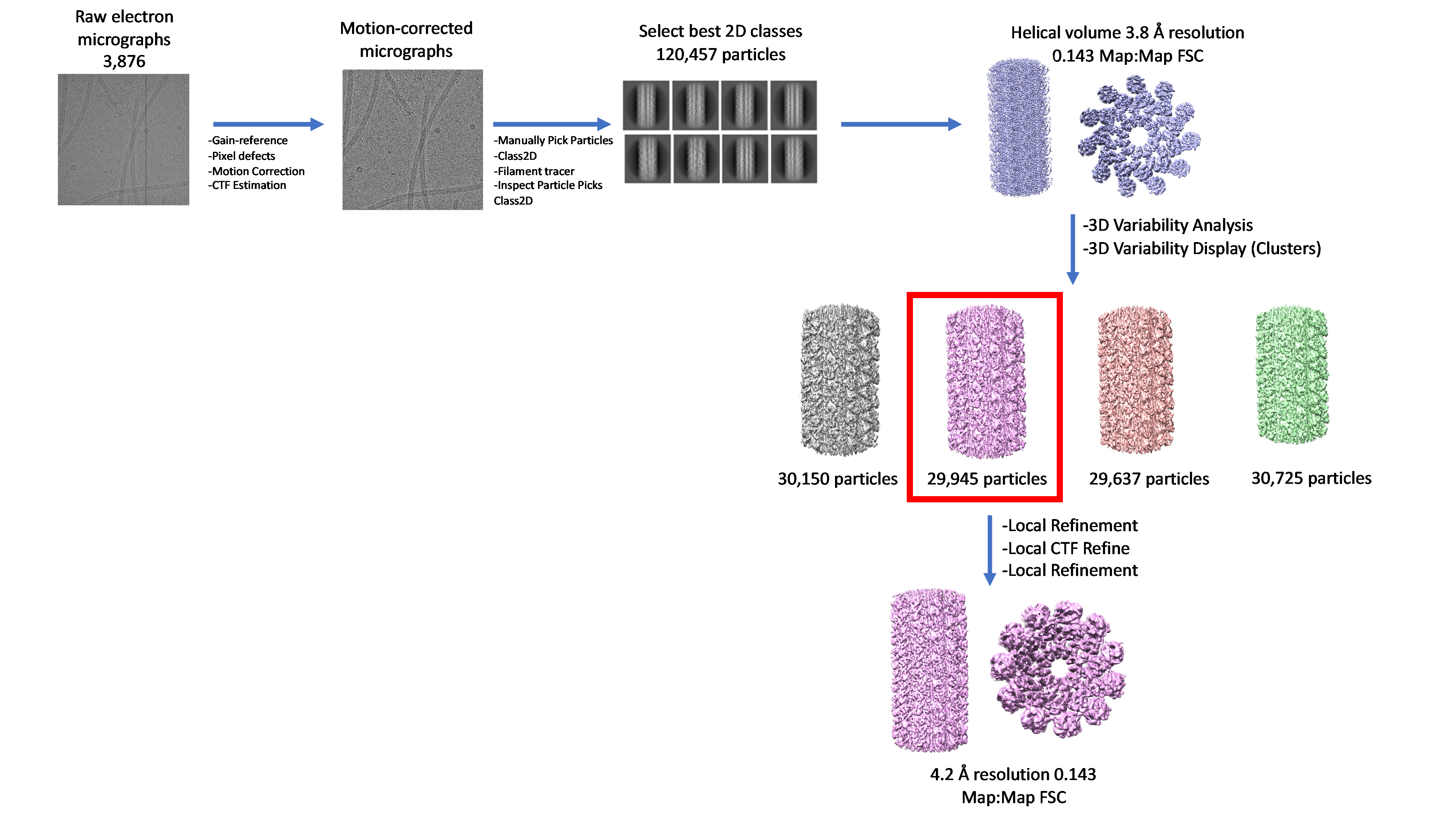

Supplement: S2 Fig — The general workflow for structural determination performed in cryoSPARC is shown here. It is important to note that the helical reconstruction was rather unusual in that an 8 Å starting D0/D1 map was required for a good final density map. For more detailed information please consult the methods section. (PNG) [file ppat.1010979.s002.png]

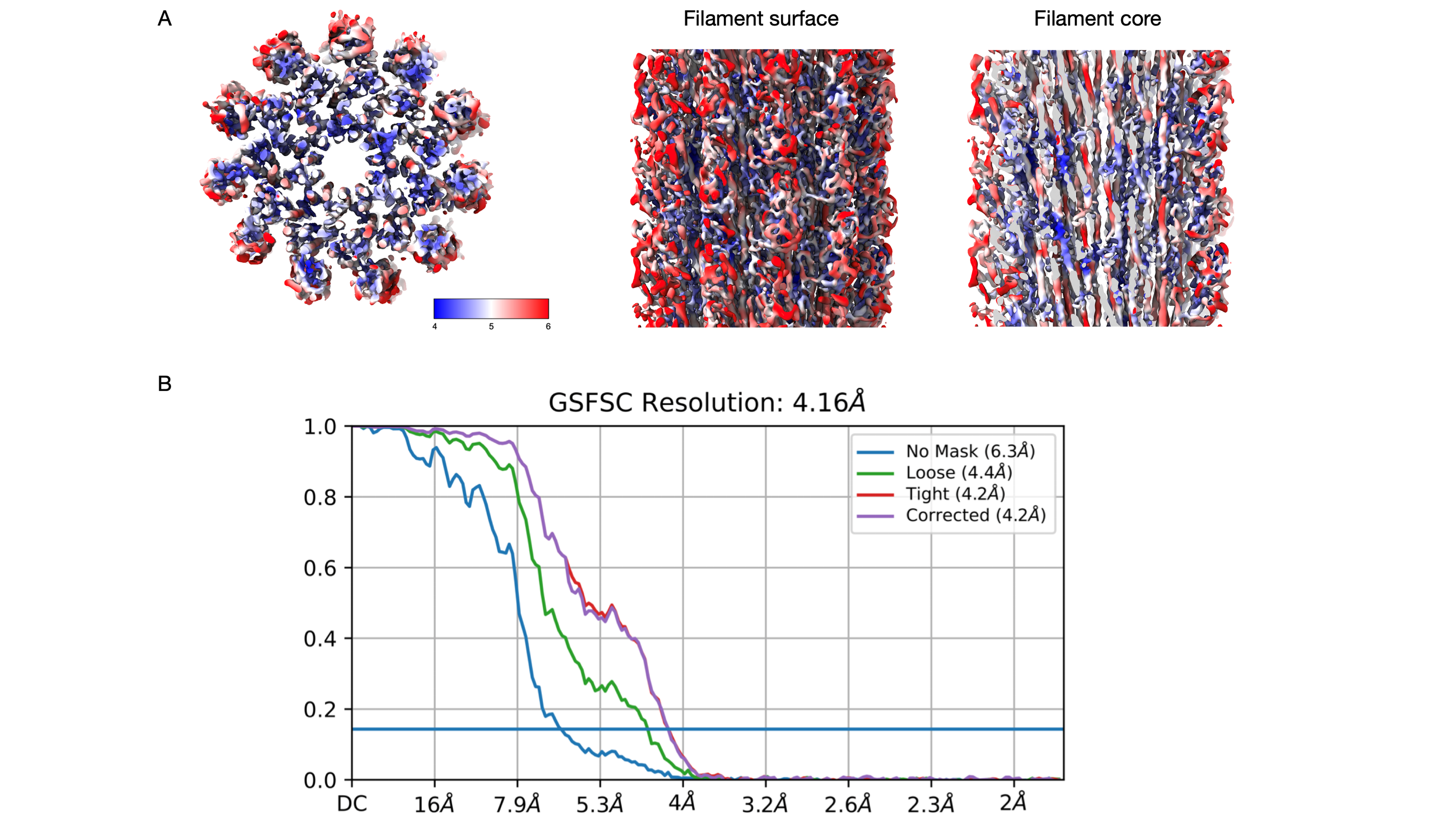

Supplement: S3 Fig — A. Local resolution estimate for the PAO1 flagellar filament. Left image shows an axial view of the filament. The middle image shows the surface of the filament. The right image shows the core of the filament. B. Fourier shell correlation (FSC) curves for the PAO1 filament reconstruction. FSC used in the curves is the gold standard 0.143 map:map FSC (GFSC). (PNG) [file ppat.1010979.s003.png]

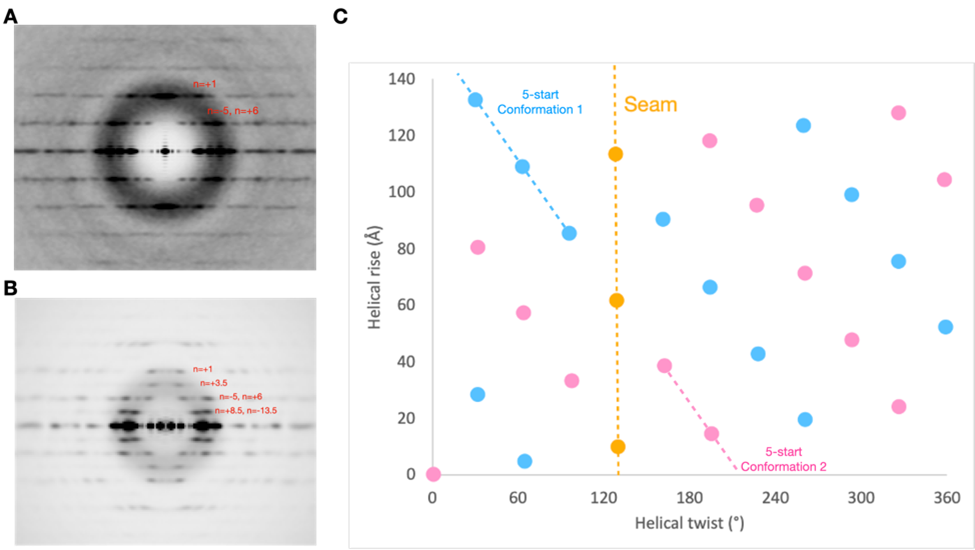

Supplement: S4 Fig — (A) Power spectrum of a standard monomeric bacterial flagellar filament from Campylobacter jejuni. (B) Power spectrum of the PAO1 flagellar filament. As shown previously [25], the number of layer lines present are double that of a typical flagellar filament, indicative of a dimerization of subunits with an asymmetric unit containing a dimer. (C) Helical net showing the arrangement of the different PAO1 flagellin conformations along the filament assuming a perfectly straight flagellum with helical symmetry for domains D0 and D1. Each dot represents a subunit and the colors correspond to the conformations shown in Fig 2. The pink and blue dashed line represents a single 5-start helix along which is broken by the seam (gold line) along the outer domains. (PNG) [file ppat.1010979.s004.png]

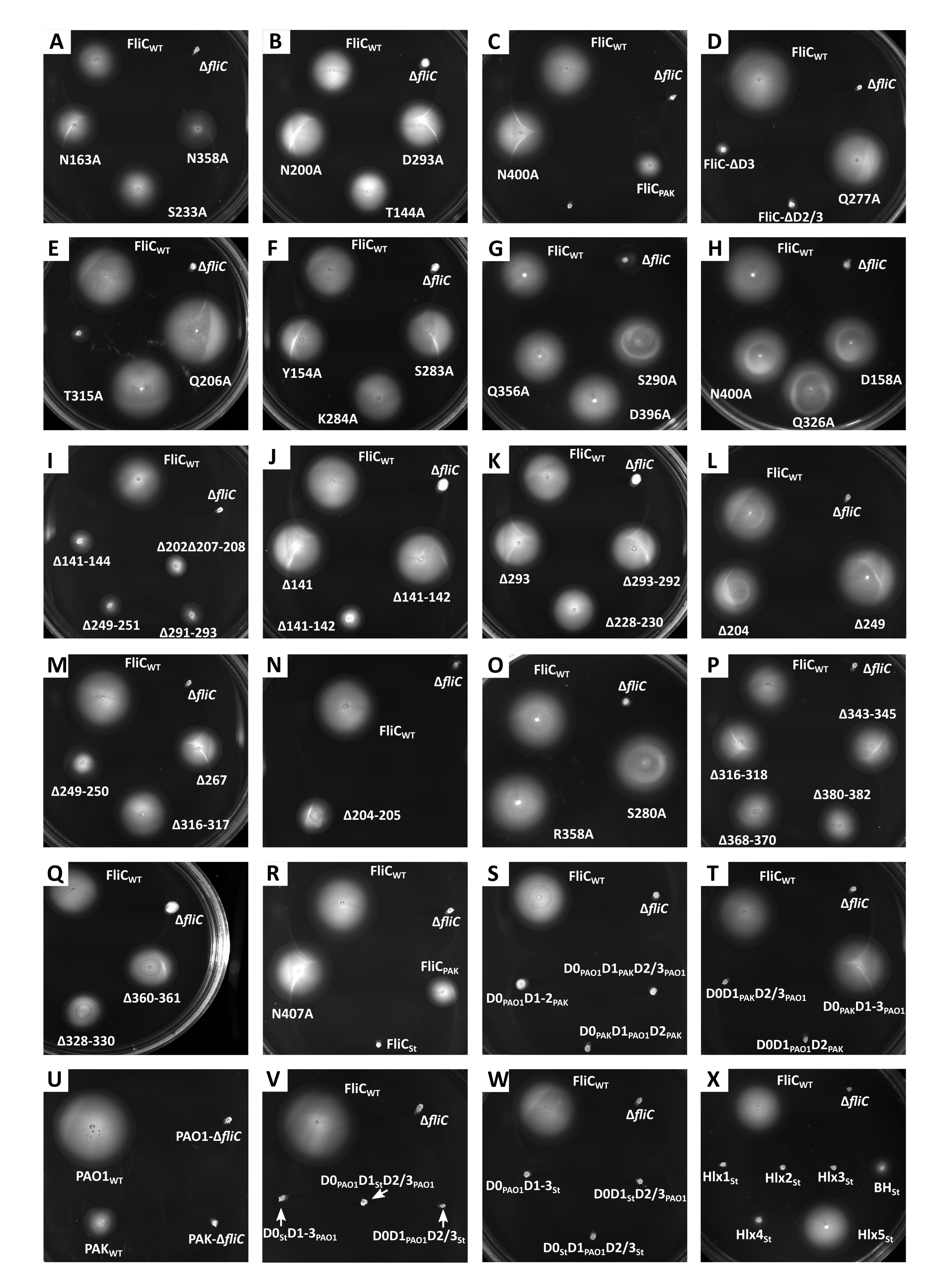

Supplement: S5 Fig — (PNG) [file ppat.1010979.s005.png]

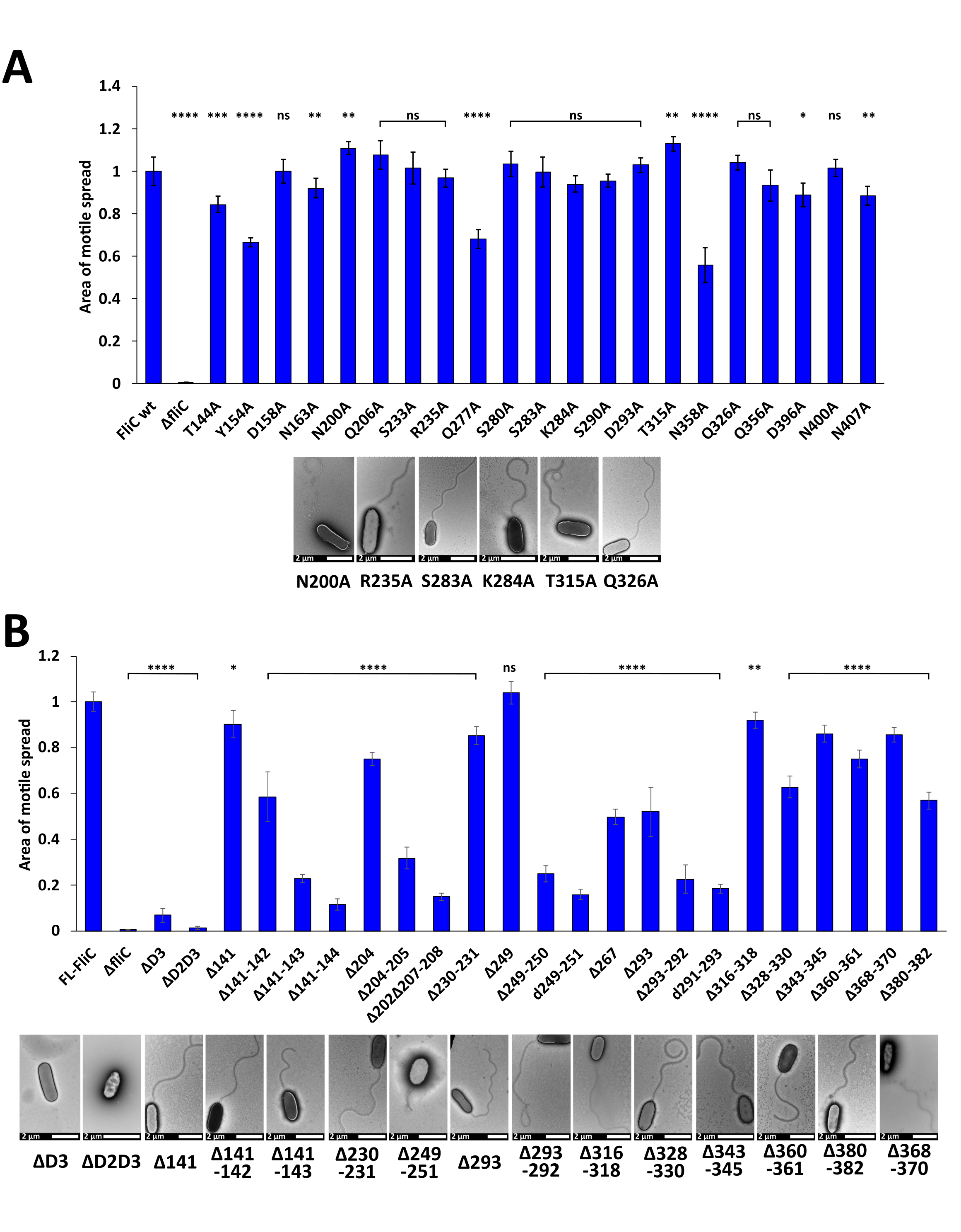

Supplement: S6 Fig — (A) Alanine mutants. (B) Deletion mutants. Negative-staining EM images presented are the images of mutants not included in Fig 3. (PNG) [file ppat.1010979.s006.png]

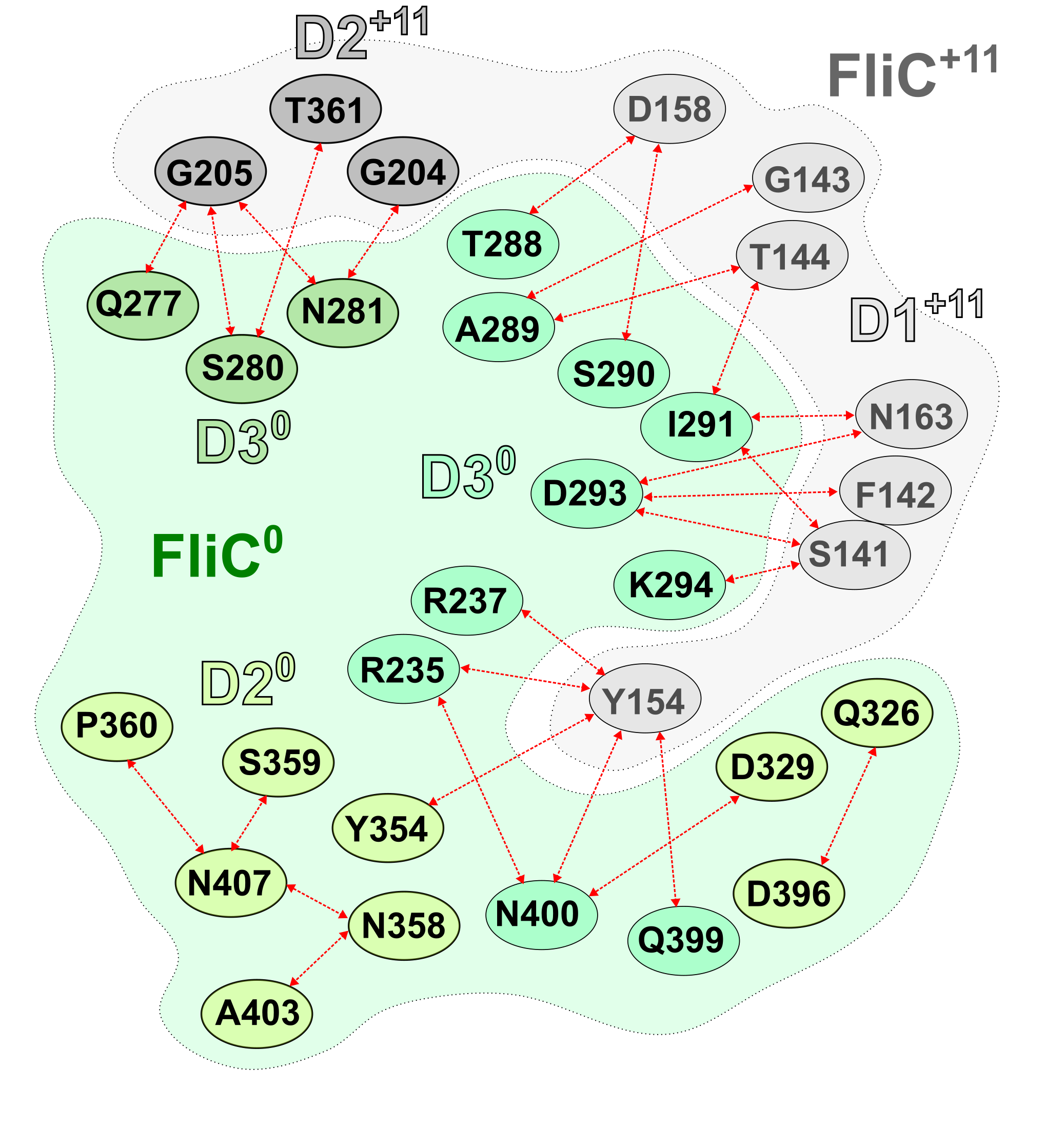

Supplement: S7 Fig — Different shades of green and gray represent different domains and corresponding residues. (PNG) [file ppat.1010979.s007.png]

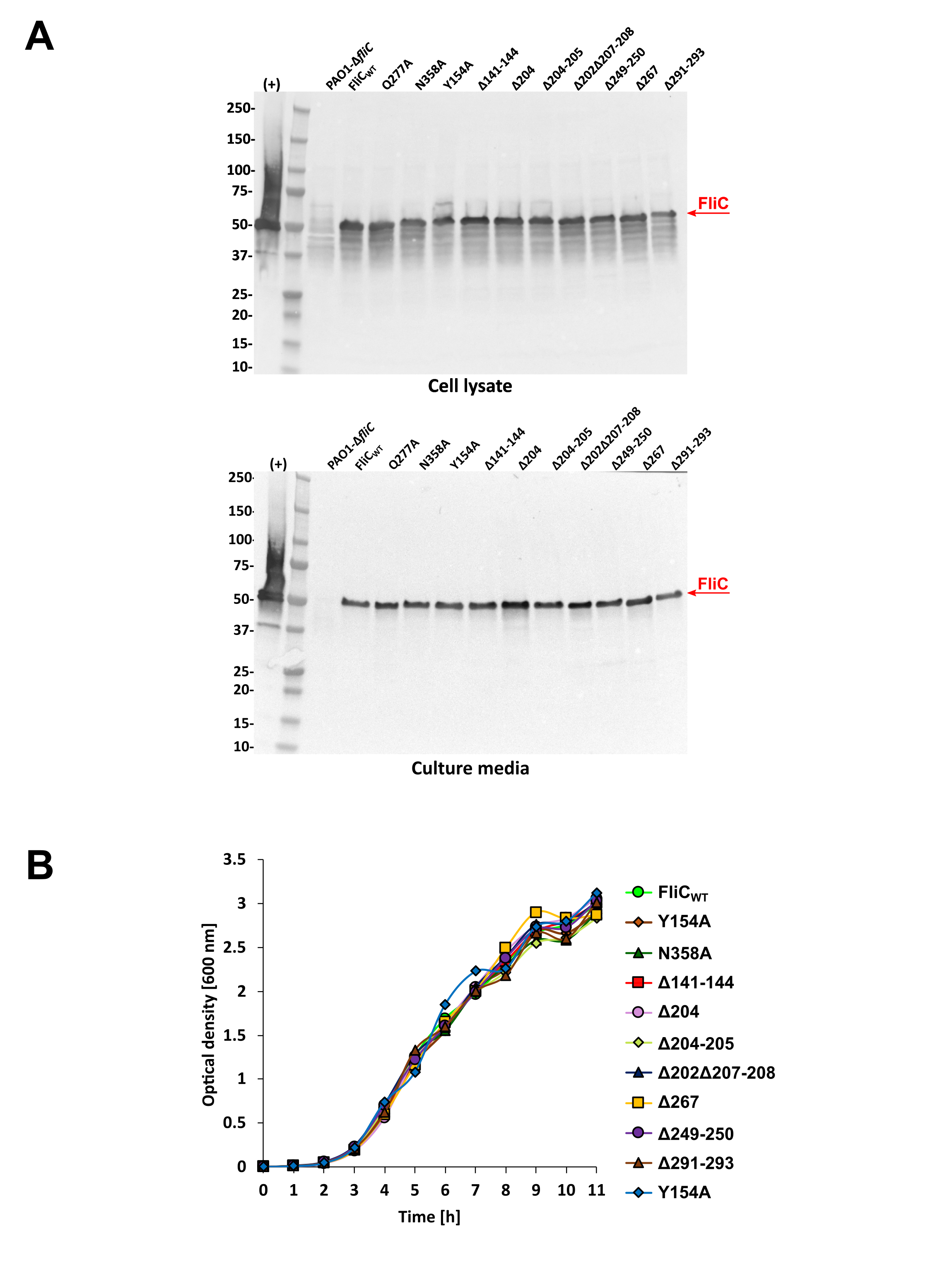

Supplement: S8 Fig — (A) Anti-FliC western blot showing the presence of FliC in cells and in the media. (+) positive control, purified recombinant FliC from P. aeruginosa PAO1. (B) Growth curves for the subset of tested mutants. (PNG) [file ppat.1010979.s008.png]

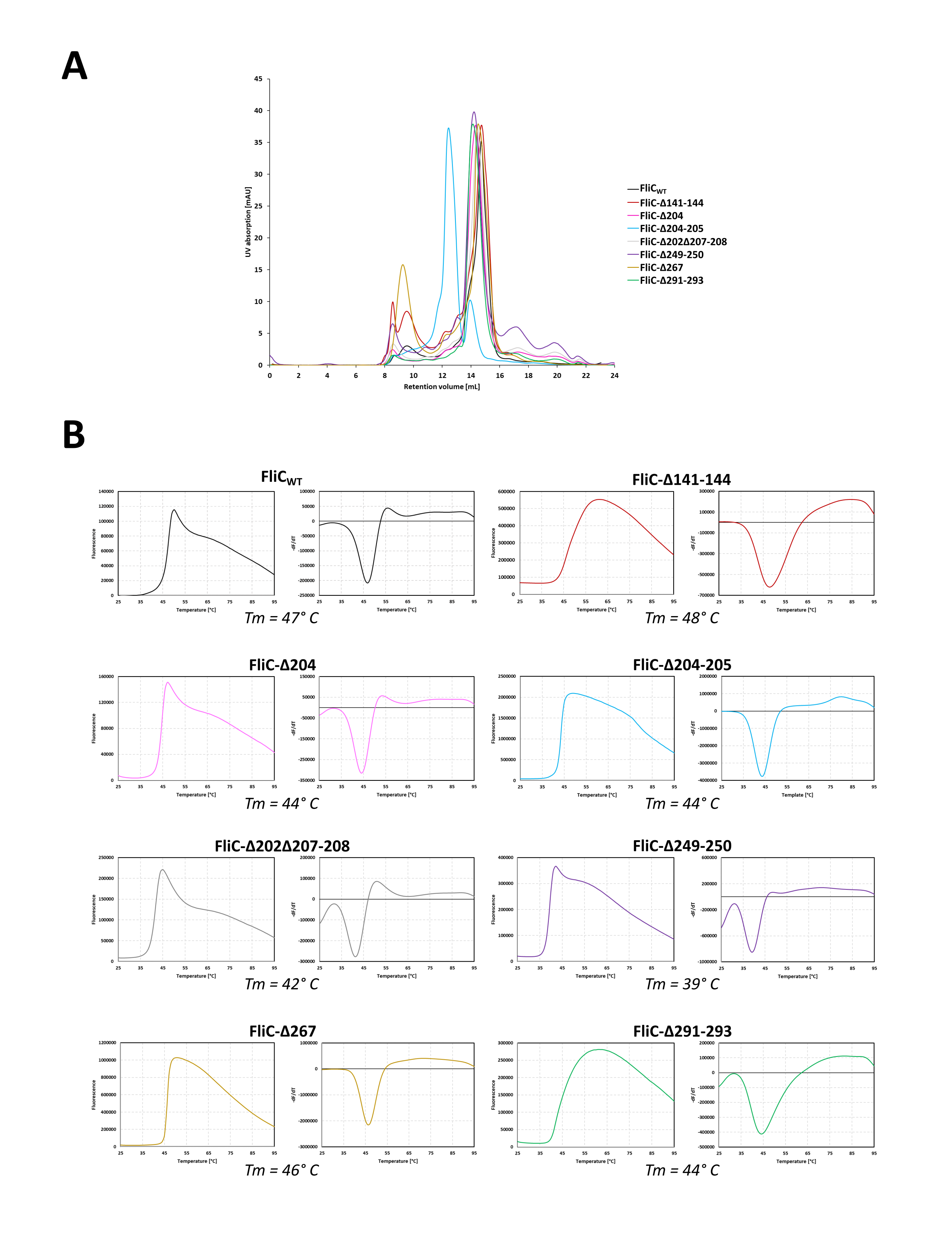

Supplement: S9 Fig — (A) Size-exclusion chromatogram for recombinantly purified wild type FliC and 7 deletion mutants. (B) Fluorescence emission and the first derivative obtained from differential scanning fluorimetry (DSF) experiment. (PNG) [file ppat.1010979.s009.png]

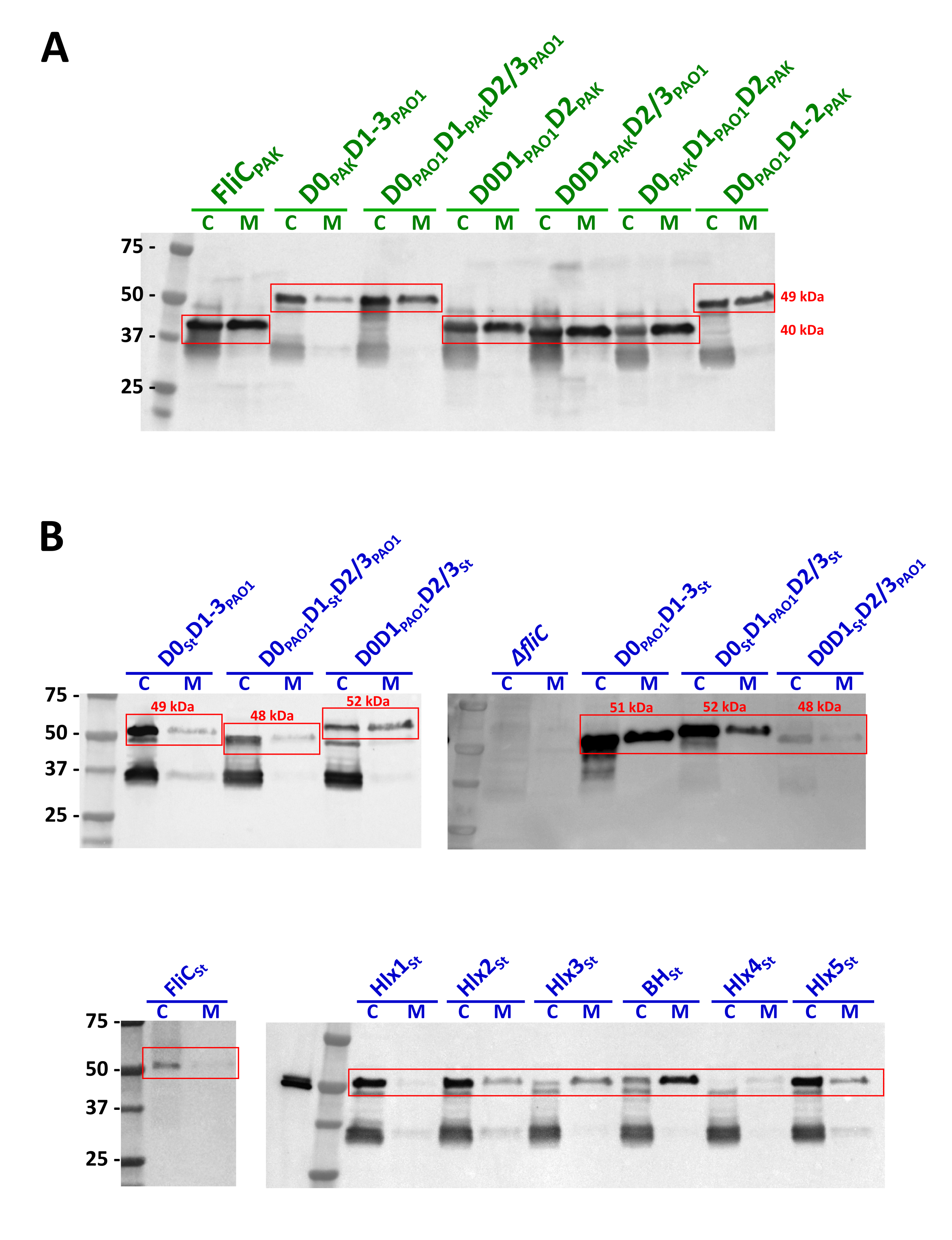

Supplement: S10 Fig — (A) PAO1-PAK FliC chimeras. (B) PAO1-S. Typhimurium chimeras. (C)–cells; M—media; St–Salmonella Typhimurium. Expected FliC band in red square. Variation in sizes is due to different molecular weight of three flagellins: PAK-FliC– 40 kDa, PAO1-FliC– 49 kDa, St-FliC– 52 kDa. (PNG) [file ppat.1010979.s010.png]

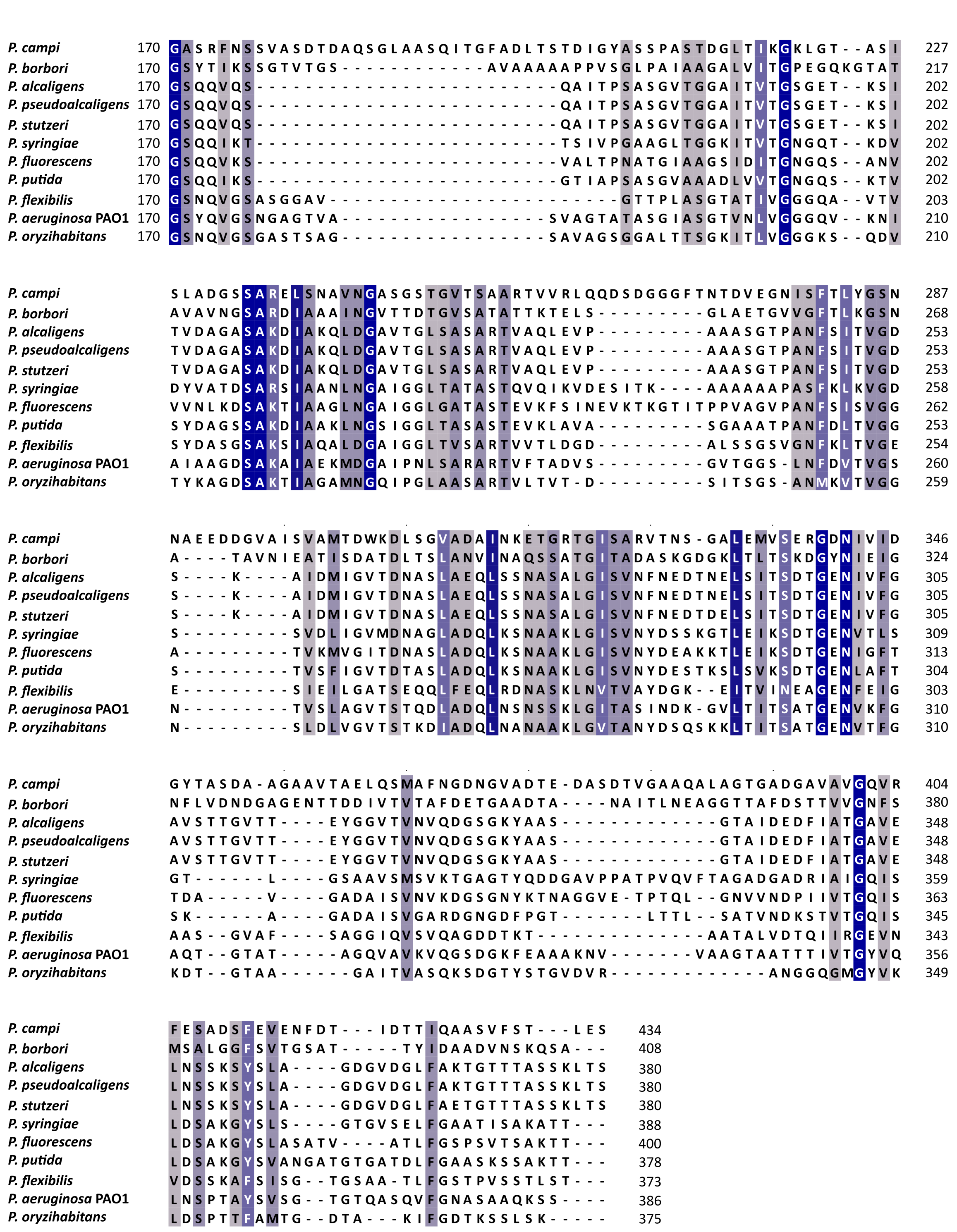

Supplement: S11 Fig — (PNG) [file ppat.1010979.s011.png]
