# Supplementary material for: An unbroken network of interactions connecting flagellin domains is required for motility in viscous environments
Source: PLoS Pathog. 2023 May 30;19(5):e1010979. doi: 10.1371/journal.ppat.1010979 (PMC10256154; doi:10.1371/journal.ppat.1010979)
Supplement: S12 Fig — (PDF) [file ppat.1010979.s012.pdf]

Aeromonas hydrophila	169	GEMEWQARSPAGSKPGAYDIA--------------------------------------D	190
Maioricimonas rarisocia		--	
Fuerstiella marisgermanici		--	
Califigura coniformis		--	
Mariniblastus fucicola		--	
Stieleria neptunia		--	
Desulfurivibrio alkaliphilus	169	-------------------------I----------GH----------------VTSSQL	177
Acidihalobacter ferooxydans	170	-----------G----ATYGDANSV------------------------------FSGTL	184
Thioalkalivibrio sulfidiphilus	170	-----------G----ARVAEGDSAL----------SG----------------DFTGAF	188
Caldimicrobium thiodismutans	170	-----------G----AHITLLPGSVG--------------SA---TGAL---PSNTNYV	194
Thermodesulfobacterium commune	170	-----------G----AHMIKTSGNKV--------------AA---SDVL---TSLTDVV	194
Desulfobacterium atlanticum	171	----------------ALVKSNNGNPLI--------TS---AN---TGTY---TGSDPTQ	197
Hydrogenoviriga calditoris	170	-----------G----AYIVSGNGGVYT--------SAADKPI---TGLE---TDKDDYA	200
Aquifex pyrophilus	170	-----------G----AYLLDLKGQSN---------SAY-DSF---ANLL---TTDTNFD	198
Persephonella hidrogeniphila	170	-----------G----AYAVKGDGQSTS--------DAT-KTL---SSLI---T-ATDFE	198
Thermovibrio guaymasensis	170	-----------G----AFMLNGQGGKVS--------DAS-KSY---ANLL---TANTGYA	199
Algisphaera agarilytica	144	------------------YL--------------------------------------DS	147
Mucisphaera calidilacus	144	------------------YI--------------------------------------VE	147
Aquispirillum sp. LM1	172	----------RS----TY-SDATGSM--------IAASAAA---------NDAATTSGNI	199
P. aeruginosa PAO1	170	-----------G----SYQVGSNGAGT-----------------------VASVAGTATA	191
Magnetoglobus multicellularis	179	-----------G----LYEVVSAGEM--------NNAGNF----------RGSIYAAIDN	205
Zobellela dentrificans	170	-----------G----LNKVELDG-------------DAVGAI-------SVGADLTGRE	194
Acidocella sp. MX-AZ02	172	--GMWATKAASG----TYKSGAN-----------ASSGE---------------VTTGGT	199
Acidovorax sp. NO-1	170	-------------------------------GNNQ-NTSVNGAG------GAASGAAWGA	191
Hylemonalla gracilis	170	-------------------------------GNNQALNTVTAAQ------SVASGATFGA	192
Massilia yuzhufengensis	170	-------------------------------GNNTLAQNTGGTMVNSVKGGAGTAGTIPA	198
Gulbenkiania indica	170	--GNNNVTVAGIKPAATS	185
Vogesella indigofera	170	--GNNNVTVAGTASKNAT	185
Azoarcus communis	170	-----------G----NYRIGSKAATTAGGTGDLTNGSTANAIA----------SNAAAT	204
Thauera humireducens	170	-----------G----NYRIGSKAATDAGAKGDLTKGSTANAIA----------SNAAAT	204
Oryzomicrobium terrae	170	-----------G----NYRIGSQAATATNSRGDLTANSSLGTAVSTAKTAVAYVAGTEPT	214
Shewanella atlantica	170	-----------G----AFQSDAP--------------GSIFGG---NLVTA-----AAGS	192
Nereida ignava	169	-----------G----VYRFD----------------STAQ-T---QGV-------TDAT	186
Desulfofustis glycolicus	170	-----------G----NY----------------QIASEV--------------TTTDFA	184
Nitrosomonas eutropha	171	-----------G----SNVLSTGG---S------HMGQAITAG---TAVPTG------AA	197
Nitrosococcus watsoni	170	-----------G----E---Q-------------LGKGIANAG---TGL--AVATDTTGG	193
Marinobacter salarius	170	-----------A----NNTLD-------------AANATLNQG---TGSTTAANATLPAQ	198
Desulfobulbus propionicus	170	-----------G----RYYVD-------------SINQTANQG---TGSTTAAGTAAPTA	198
Nitrospirae bacterium CG 4 9 14 0 8	170	-----------G----NNSVTGDSADVS------GINSAVAAA---ANVNGGSGSTVTTA	205
Rhodobacter CACIA14H1	170	-----------G----AY----------------KVKSEVS-------IA--ASGG----	185
Cereibacter sphaeroides	169	-----------G----AH----------------KISSAST-------VV--ADAALTDT	188
Roseovarius mucosus	169	-----------G----AH----------------TVTSNVS-------LA--AGAGTADT	188
Bradimonadales bacterium 2099667	171	------------------------AS--------LSGQ-QN----------------RIN	181
Myxococcales bacterium 2026763	173	---AIKQ-TAVV----------	180
Bordetella SCN 67-23	173	---------------------------------------YNGFNA-TNLSI-----GTAS	187
Legionella pneumophila	173	------------------------AT--------ATGTEVAGAAA-TDIT----------	189
Geothermobacter ehrlichii	173	------------------------AK--------GTGSEVSANNA-TDIT----------	189

Aeromonas hydrophila		LKLSSQPV-----------TLNGVTFDKDYS-----------------------TLEAFV	216
Maioricimonas rarisocia	145	--------------------VTGTATDTDIS---------------VLKATADTAAGTYA	169
Fuerstiella marisgermanici	145	--------------------VKATSTDSAVT---------------FLKGTSDTTAGTYT	169
Califigura coniformis	145	--------------------ISGVSTDTDVT---------------VLKATGDTKAGNYA	169
Mariniblastus fucicola	145	--------------------VSGVADDPDVT---------------YLKSTAQTSAGTYA	169
Stieleria neptunia	145	--------------------VTGTTDDTDVT---------------FLKGGSNTSDGTYT	169
Desulfurivibrio alkaliphilus		NFSEAGTSSLSLYSSIENRTYNLNTVD--VR---------------YDN-SRENSLGAVA	219
Acidihalobacter ferooxydans		SSSTSGSFT-----------INGTKGS--AG---------------IST-TAGESLSNVI	215
Thioalkalivibrio sulfidiphilus		DFGTPGTLA-----------IQGVEID--LA---------------DLN-TDATSLTEVV	219
Caldimicrobium thiodismutans		SSS-NVTA-----------SVGGRVINAT--IQN--------------GTNYIYDAGYVA	226
Thermodesulfobacterium commune		FGT-NQTI-----------TVGGRVIDAT--IQNVNGS----------TTNYILDAGYMA	230
Desulfobacterium atlanticum		FNS-GEDL-----------IINGTSVV--------QGF----------ADGDDLDAAKIA	227
Hydrogenoviriga calditoris		LAN-GEEL-----------RVGGQVVL--------QGT----------ATTVLVDAATAA	230
Aquifex pyrophilus		IAS-TETI-----------SIAGVTHS--------PP------------TNIVTDARYIA	226
Persephonella hidrogeniphila		YNSASDTI-----------TVQGVDLS--------AAF----------TSGQMIDAKTLA	229
Thermovibrio guaymasensis		YNSSNDTV-----------KIEGVDLS--------GAF----------NDGGAVDAKALA	230
Algisphaera agarilytica		GVVTSQ-------------------------ISDINVF-----------G-A---NFGLD	167
Mucisphaera calidilacus		NLTTSQ-------------------------ISDVSVY-----------G-A---NFGTN	167
Aquispirillum sp. LM1		NRMQAQTL-----------TIRSKGQTGLVDLG-------------GGATINGLSAKEIA	235
P. aeruginosa PAO1		SGIASGTV-----------NLVGGGQVKNIA-----------------IA-AGDSAKAIA	222
Magnetoglobus multicellularis		GVTSLDSV-----------TIQGFMGAEYIE-----------------IS-DYSSAKQIA	236
Zobellela dentrificans		AFGDGGKV-----------TITGSAGSATIT---------------LPAAADNAEANDVA	228
Acidocella sp. MX-AZ02		GAFTAGSL-----------SITGSNGATSTL---------------SGIT-ATESAASLA	232
Acidovorax sp. NO-1		NSTTSGTV-----------AINGSLGSKTVN-----------------IA-DNATAKTIA	222
Hylemonalla gracilis		NGMSSEAF-----------TINAAAGSAAVN-----------------VA-ANETAKSVA	223
Massilia yuzhufengensis		NNVAGGAI-----------TVQGNGTSMSYT-----------------AV-AGDSGKKIA	229
Gulbenkiania indica		AVQAGKDV-----------TIAGSLGSAKYT-----------------TV-AGDSAKTIA	216
Vogesella indigofera		NVVAAKDI-----------TVAGSLGSAKYT-----------------TV-ATDSAKTIA	216
Azoarcus communis		SRVAGGSI-----------TISGATGSATVS-----------------IT-AGDSAKKAA	235
Thauera humireducens		SRVAGGAI-----------TISGATGSATVT-----------------IA-AGASAKKAA	235
Oryzomicrobium terrae		SAIAADTV-----------TINGAVGSKTVT-----------------VS-AGDSAKTVA	245
Shewanella atlantica		NGTTAGDI-----------TITQ--GTAVTA---------------VTLA-ADDTGTEIV	223
Nereida ignava		SDKIVTDF-----------DILGKDGAAEAA-----------------FA-AGSTAKTIA	217
Desulfofustis glycolicus		ATFGAQSL-----------EISGNQTTETVT-----------------TA-ADASAKDIA	215
Nitrosomonas eutropha		AVVATDNF-----------TLTTAQGGTTAA---------------IIYD-VSAGANGIA	230
Nitrosococcus watsoni		NTVAAQNI-----------TVNGSTGSKSVA-----------------LT-GNESAKAIA	224
Marinobacter salarius		NTIATQNL-----------TISSSLDSQVVP-----------------IT-AGDTAEDIA	229
Desulfobulbus propionicus		NTVAGQDL-----------TVTGSTGSTTIT-----------------IG-AGATAWDIA	229
Nitrospirae bacterium CG 4 9 14 0 8		NRITAQTL-----------TISGNLGQDTVA-----------------VT-LGESAADIA	236
Rhodobacter CACIA14H1		-VTTGKQL-----------IVSGHAGNAEIE-----------------TT-ADMSAKQLA	215
Cereibacter sphaeroides		TIAASTDI-----------TITGFAGSDKIT-----------------TA-AGDSARTLA	219
Roseovarius mucosus		GIAAATTI-----------NITGFAGSAELT-----------------TT-AGQSAEEMA	219
Bradimonadales bacterium 2099667		DDAMGNEI-----------TLGTGNRQASVTLSENNGFDTSSPD----NSARDYSAIAVA	226
Myxococcales bacterium 2026763		---STNAL-----------SKN-GGDI---LINSITVRTTSAADDQLSTSFAAGSALAKA	222
Bordetella SCN 67-23		DTAAAQSV-----------TIGSAGTF---AL-----------------GSIANDAKAIA	216
Legionella pneumophila		-------I-----------AIGGGAAT---SINSSANF----TGALN--GQDATSAYAKA	222
Geothermobacter ehrlichii		-------I-----------SVGGGATY---NIANSANF----AHSTDTTYRGGDSAYAKA	224

```
Aeromonas hydrophila                 ADINGTTFPNNEGPVKAQ-QLPFSVASAVDLSAL-PSYIDVAGHSVDLTTGIDMTGWD--  272
Maioricimonas rarisocia              VSVGTR--AERAY-VDTT------------TA----LAAGTLAQDETLT----IND----  202
Fuerstiella marisgermanici           TTVTTA--AERAN-VSA-------------TA----QTTTLNTDETLT----VND----  200
Califigura coniformis                VSVTTA--GERAN-VEAG------------TV-----QTANLAAEETLV----VNG----  201
Mariniblastus fucicola               VEVTTQ--AERAN-VTAG------------TA-----QSGNLAADETLT----VNG----  201
Stieleria neptunia                   VEVTTQ--GERAN-ITAG------------TS-----QTGNLAADEVLS----VNG----  201
Desulfurivibrio alkaliphilus         DEINKL--SDQLG-ITAN-AVVR-----TTTD-----ENIEKGQTGVFS----ING----  257
Acidihalobacter ferooxydans          ASINNA--SSQTG-VYAQ-TASD-----VSGT----LSYTSSGGATTQT----ING----  254
Thioalkalivibrio sulfidiphilus       NRINAL--SAETG-VTAA-LSSQ-----AEAT----FAITAADAAGTLN----ING----  258
Caldimicrobium thiodismutans         EQINRN--LGDIG-FVAK-AVNI-----SIGNKYTALA--DISTSATVTFF--VGD-KS-  271
Thermodesulfobacterium commune       EQINSN--LSDIG-FKAK-AINV-----SIGEKYTAIA--DVSDTATLTFY--VGD-KS-  275
Desulfobacterium atlanticum          QNINEN----VKG-VTAT-AKNE-----ITGTVEFGTISIGSDDSATIT-I--SGP-E--  270
Hydrogenoviriga calditoris           NNINNDATLQAAG-IEAV-AKNK-----STGADFTGVVSGDAGDTVTLHFY--VGA-RDI  280
Aquifex pyrophilus                   DWINGDPTLQQMG-IKAK-ASNR-----VVGDPWVNVAV-DTGDSLTIKFY--VGT-ST-  274
Persephonella hidrogeniphila         DAINKNADLQAKG-IEAS-ATNK-----SVAGTAFSTVS-IGTGGLTIDVY--VGP-ET-  277
Thermovibrio guaymasensis            DYINSSEELKEKG-IEAM-AFNK-----SVADTNFSDIS-VGTSGLTIKFY--IGN-KNG  279
Algisphaera agarilytica              DNINVQ--VE----VINSAEKGT-----LFLSGNTGAPGALLSSVSFE----LQG--S-  209
Mucisphaera calidilacus              SSVPVS--VE----VLASAQKAT-----LFLSGYTGTIPGD----VVFT----LGG-T-  205
Aquispirillum sp. LM1                ARVNAQ--STYTGGVAAR-AETY-----AFLSLG-TQS--TMQASVNFK----LNG----  276
P. aeruginosa PAO1                   EKMDGA--IPNLS-ARAR-TVFT-----ADVSGV-T------GGSLNFD----VTV--G-  259
Magnetoglobus multicellularis        ESVNAK--SDLTG-VEAK-AGTY-----AKIYNL-SR-----TGDLTFK----LHG----  273
Zobellela dentrificans               AAINAQ--SSVTG-VTAE-ATNK-----VEITGL-AGI--DAGENVTFT----LNG----  268
Acidocella sp. MX-AZ02               TAVNDT--SSTTG-VQAQ-ATNT-----ITLKAA-AG---GGSGNYSFK----IGAATG-  274
Acidovorax sp. NO-1                  GNINAV--QADTG-VSAT-ARTT-----AQVS-F-GA-----AGSYTLA----LRS-DN-  260
Hylemonalla gracilis                 VKVNQV--TPATG-VTAE-ARTN-----LLMT-F-QS-----VGAHTLT----IQS-DN-  261
Massilia yuzhufengensis              DAINAA--SGNTG-VSAS-ASTS-----ATLGGF-AA-----GT-FSLT----LQGAPK-  268
Gulbenkiania indica                  AGINRL--TEQTG-VTAS-ARTE-----ANLS-L-VA-----GKTYAID----ITS-DN-  254
Vogesella indigofera                 ANVNKL--TSQTG-VTAS-AKTE-----SNLT-L-AA-----SGTFAVN----ITS-DN-  254
Azoarcus communis                    ELINAQ--TSTTG-VKAS-AKTE-----IDVTAM-AA-----NTTYKFD----VSS-NN-  274
Thauera humireducens                 EQINGE--TGKTG-VTAS-AKTE-----IDVTAM-AA-----NTSYKFD----VTS-DN-  274
Oryzomicrobium terrae                ANINKA--TGNTG-VTAS-AKTE-----IDMTGL-TA-----GASYKLD----VYS-DN-  284
Shewanella atlantica                 SKINNA---GTG-VKAI-AETN-----IQLDLT-AAF----DSSMIMS----VDD--G-  260
Nereida ignava                       AAVNAD--SSGTG-VTAE-AVTK-----ARISLD-AV----PTSTVTFN----LNG--G-  256
Desulfofustis glycolicus             DAVNTK--KVETG-VEAE-ATTN-----LKVDNL-AS-----SGSVSFS----LKG-AN-  254
Nitrosomonas eutropha                AAINSA--GTDVG-LSAV-ATNS-----ATLGKL-AS-----AGTVSLT----L------  265
Nitrosococcus watsoni                DLVNGQ--SGATG-VTAS-AQTS-----VKLDNV-AD-----DGTVSFT----LQS-SG-  263
Marinobacter salarius                AAINDI--GATTG-VNAT-ARTS-----ATLSNT-ATTPIAVPQTVSLT----LSN--G-  272
Desulfobulbus propionicus            SQVTNA--ADKTG-VTAT-ARTE-----VSLSGL-SA-----DGVVTLT----VGS--G-  267
Nitrospirae bacterium CG 4 9 14 0 8  TGINDK--TSTTG-VTAT-ASTV-----ATLSTI-ST-----G-ATTFK----IAG-DD-  274
Rhodobacter CACIA14H1                AEVNAK--SAQTG-VQAT-AVSK-----AKLSGL-SD-----VGKISFE----V------  250
Cereibacter sphaeroides              ESINKK--TSSTG-VEAT-ATTK-----AQLSGF-TK-----GDTVSFK----IGT-AD-  258
Roseovarius mucosus                  SAINDV--SASTG-VTAS-ATTN-----LELSGF-SA-----ADTVTFD----V------  254
Bradimonadales bacterium 2099667     AAINSI--ANVTG-VQAS-VKES-----VFQL-N-NLSLYSLGQTEAVV----SGD----  267
Myxococcales bacterium 2026763       AAINDA--TNYTG-VSAR-VLAT-----EARTDL-DIAGGSLTNTSFIE----ING----  264
Bordetella SCN 67-23                 NAMNTQ--G-IAG-LNVT-ANAT-----E-VAGT-STVTTTAGTASFT----LNG----  256
Legionella pneumophila               AAINDA--G-IGG-LSVT-ASTS-----G-TQAV-GAIGGTAGDTYNLT----ING----  262
Geothermobacter ehrlichii            AAINDA--G-IAG-LTAT-ASTS-----G-NTTF-AAVGGTSGDTYTLK----VNG----  264

Aeromonas hydrophila                 ---PVNP-------TAKNSAIMATVVQRINSTAQAAGLGFYVSASNNSNPASNALTMHSA  322
Maioricimonas rarisocia              ----VSI-------TLNSGLTRVEVQNRINEFTAQTGVVAEDD-G-----AGTGIRLRTE  245
Fuerstiella marisgermanici           ----VEI-------TLTAGLTQNQVVDRINEFTTQTGVKAEIN--------GTTTRLYTE  241
Califigura coniformis                ----VSI-------TLAAGLDKTGVISRINEFTDQTGVVADAN-G-----TGGATRLYSE  244
Mariniblastus fucicola               ----VSI-------TLNSGLDQAGVIARINEFSGETGVIAEDDPA-----SAGQTRLYTT  245
Stieleria neptunia                   ----VSI-------TLNSGLSRAGVVARINEFTDQTGVVAEDV--------SGGTRLRSI  242
Desulfurivibrio alkaliphilus         ----VNI----GSISVQENDADGALVAAINQKSNQHGVFASVDFE--------GKMTLTS  301
Acidihalobacter ferooxydans          ----VQV-------SISANATLSQAVDSINAYSAQTGVTATASGS--------SGIVLSD  295
Thioalkalivibrio sulfidiphilus       ----VNI-------AVDSGDTAELLAGKINALSNQTGVTASFDGA--------DLTLT-S  298
Caldimicrobium thiodismutans         ------FNF-----TADTTITLDQLVEKINRAAATAQADLSASV------SDGRLVLTSS  314
Thermodesulfobacterium commune       ------FSF-----TAETTISLDELVEKINREAATAGADLTAST------DAGRLVLTSS  318
Desulfobacterium atlanticum          --GNATINL-----ASGQGYDLNAMISLINGVSNNTGVFAKADS------TGTKLVLYTQ  317
Hydrogenoviriga calditoris           TSPNFSITG-----VTTTTTLSDLVTQINSQASATGTPITARA------ENGKLVLETT  328
Aquifex pyrophilus                   -TPAITLTY-----GPGKTITLDRIISDINSKA--TGLNLVAKE------ENGRLVIET-  319
Persephonella hidrogeniphila         -TADFTLTY-----ASGTSLSLTQLIDDINTKAKENNLNLVAKD------EGGKLVLET-  324
Thermovibrio guaymasensis            SSADITLSY-----ASGTTLTLDKLIADINSAAQNAGVELTASK------SGERLVLST-  327
Algisphaera agarilytica              -RGVQVF-------NFASGTALSAVAAAVNNVRDATGVTARLASATN---QTSGLLFESE  258
Mucisphaera calidilacus              -GGAQTL-------EFASGQDMSDLMAQINSRSDATGVAARLFDPLN---SAAGIVLEST  254
Aquispirillum sp. LM1                ----QTI----SAYSSSTASDVDGLVTAINDVSGKTGVVAQKVDLPG-SAGTYRVQLFAA  327
P. aeruginosa PAO1                   -SNTV---------SLAGVTSTQDLADQLNSNSSKLGITASIN-------DKGVLTITSA  302
Magnetoglobus multicellularis        -SSEVDI----SA-TIESKSDLTPLYEDLESKAAASHITPKLSA------DKSSIYLYAE  321
Zobellela dentrificans               ----ENVSY-----NASNANSLAELASAINGKSSATNVSAEVITKDD---GTSALVLTSA  316
Acidocella sp. MX-AZ02               -AGTSSAS-AVGKAVTVNASSLSELVSQINGGTTTNGISATLNS------AGSAVTLTQA  326
Acidovorax sp. NO-1                  -SADLNVSFTLSA-SNT-SEGLTAAVSAINEQSSKTGVTATVNA------AGTAIELTNA  311
Hylemonalla gracilis                 -GTPEPISFSISN-PGT-PDGLSNAIAAVNEKSSKTGVIASLNA------AGTALVLTNI  312
Massilia yuzhufengensis              -ADGSANPVTVSA-TLTGANDLSGLTKAINDQTGATGITAVADL------STGKIALTQS  320
Gulbenkiania indica                  -STARTVSFSTGA-TLNSADDYSEAINAINAASSKTGVTAEYDS------KLGGIKLTNA  306
Vogesella indigofera                 -ATAVTVSFRTGA-SVSSADDYSEAINAINAASGKTGVTAEFDS------SLGGIKLTNA  306
Azoarcus communis                    -STAVTLSFTVGS-AVD-QDGLTSAVNAFNDVSSKTGVTARIND------TNDGITLLNA  325
Thauera humireducens                 -STAVTISFTLGA-SVD-KDGLASAVNAFNDVAAKTGVTARVND------GGNGITLLNA  325
Oryzomicrobium terrae                -TTASTISFTVGS-AVN-ADGLSAAVNAFNDVSSKTGVTARVND------AGNGITLLNS  335
Shewanella atlantica                 -TSTSSL-------NLTGIANNTDLATAINDVSGETGVTAKI--------DNGTLTVTST  304
Nereida ignava                       -GTAAAV----SA-TVVSNTDLTSVLDAINALSGTTQITATFDGT-----DKSKLILSDA  305
Desulfofustis glycolicus             -STAVKV----SA-VITDNTDLSALVSAINDNSGATGITARLD-------NDGAMIMTSS  301
Nitrosomonas eutropha                --NDQAI----SA-NVASTGDLSALAAAINGVAGTTGVSASFANP-S----NLSEITLSTT  314
Nitrosococcus watsoni                -GSAAQI----SA-GVT-TTDVTNLADAINAQSAETGVTATLSE------NRDSITLENA  310
Marinobacter salarius                -SSSATI----SA-QITDANDLSAIAREVNAASGKTGITAEVA-------NDGSITLIQE  319
Desulfobulbus propionicus            -GNTATI----NA-SVT-TTDLSELATVINDQSGTTGVTATV--------DGGTINLVQA  312
Nitrospirae bacterium CG 4 9 14 0 8  -GTLVSI----SASTTGSGTGLDPLVQAINDVSSTTGIVAER--------SGDSITLTNS  321
Rhodobacter CACIA14H1                --NGQNI----GTVAISNTDDLRSLRDAINTKTTSTGVTATMGA------DNSEIILTDS  298
Cereibacter sphaeroides              -GNEVSI----GDVSITDASDVRGLRDAINAVSGQTGITAAMAKD-----DNSKIVLTDA  308
Roseovarius mucosus                  --NGVNI----GTVAISDTSDLRGIATAINNQSGRTGVTATMGD------DNSSIKLTDA  302
Bradimonadales bacterium 2099667     ----LTING--QE-IVGSVGSIDGLVNTINNFSSVTGVEARLN-------GSNSVELFAR  313
Myxococcales bacterium 2026763       ----VTLT---TF-DVATDDADHELKNQINAVADRTGVLASYD-------ENSRLILTAG  309
Bordetella SCN 67-23                 ----LTINL--TA-TTNAATNRENALTAINAQSATTGVRATD--------TGSGLKLEAA  301
Legionella pneumophila               ----VAIYT--NL-DVATALTNSDLRDAINGVSNQTGVVASL--------NGGNMTLTAA  307
Geothermobacter ehrlichii            ----VTVYN--NA-DVSTALSVTDVRDAINAVSGQTGVIASS--------DGSTLTLTAA  309
```

Aeromonas hydrophila	Q-GNSLALANGTTAPPQPAPP----------ALPVSS----NSSLSA-------------	354
Maioricimonas rarisocia	AFGTAASISVVSNQSGA-NSA-GFT------TSEQT----DT---------------GVN	278
Fuerstiella marisgermanici	DFGSDAEISAISNVAASGSSS-GIG------TSALT----DD---------------GVD	275
Califigura coniformis	AFGSDAEISVISNTAGAVDSS-GLG------TTELT----DT---------------GVD	278
Mariniblastus fucicola	QFGADASISVVSDTAAAATSS-GFG------TTADT----DD---------------GVN	279
Stieleria neptunia	EFGSDAEIEVVSDTAAATTSS-GFG------TTLDS----DQ---------------GVN	276
Desulfurivibrio alkaliphilus	--MDGRAISVTADDATRGVLG-GTEDMSTLGHIQLTQ-MGAA---------------EIM	342
Acidihalobacter ferooxydans	--NTGASITFSGSSTNITASG-AAT-TATSGTFQ-------A---------------GVE	329
Thioalkalivibrio sulfidiphilus	--NGDITIERTG--GGALVIG-GLG-AGESGTIM-------R---------------GID	330
Caldimicrobium thiodismutans	K-GYTIGVQVAL----SGTTA---------GTINLDQ-LIQSA-----------------	342
Thermodesulfobacterium commune	K-GYTIGVEISL----ASA-T---------GTININQ-IVNDLE---------NASAG--	351
Desulfobacterium atlanticum	N-GGTFNLDFSVSPGAT-SGN---------TSIDLAK-FGAATS---------TVV----	352
Hydrogenoviriga calditoris	N-GETIAIEAEVTADAGSNNN---------VAVNFSQ-LIEGAS---------DVT----	364
Aquifex pyrophilus	S-GETVGVEVSK----SGTGG---------TTFSLDQ-IISGVN---------KTV----	351
Persephonella hidrogeniphila	E-GETIALEVTA----G--SG---------TTIDLGT-LIEDAS---------GSV----	354
Thermovibrio guaymasensis	D-GSTIGLEVVA----G--SG---------TTVDLST-LLEGAT---------GSV----	357
Algisphaera agarilytica	GYGTSSFVSVR-------------------------K-LDD-GDFFDTFDAQGGS----Q	287
Mucisphaera calidilacus	TYGSDAFISVD-------------------------V-RDDPASIFSPLMSAAGT----Q	284
Aquispirillum sp. LM1	D-GSDIKIQEASVTSSNTAGA---T-WGNASIMTLQGALDSNGTITPNAGTAASHTIGQV	382
P. aeruginosa PAO1	T-GENVKFGAQTGTATAGQ-------------VAVKV-QGSDGK-------FEAA--AKN	338
Magnetoglobus multicellularis	A-GENIGIEDFYNPTMPT-------------VMNFVG-LEPDGK-------TTT---GVS	356
Zobellela dentrificans	N-GEDIALSDFSGADGD--AA---------ETFNVAK-VNLDGV-------AGIPA----	352
Acidocella sp. MX-AZ02	N-GDNIAITAAATSATSK------------------G-LTTTGA-------TA------A	353
Acidovorax sp. NO-1	T-GNDIRVADTAVQNAGA--------------VTVTK-LQADGT-------AVA---GDV	345
Hylemonalla gracilis	T-GNDIMVSDTSVINAGD--------------VTVAK-MRPDSS-------GALSAVSTM	349
Massilia yuzhufengensis	Q-GYNIGLKNNGTQ--TT--------------ITMTG-AAGSEG-------GSG--TPVT	353
Gulbenkiania indica	T-GADIQLASAAGS--GD--------------FTLSG-YDNSGT--------TP--ALVA	338
Vogesella indigofera	T-GSDIQLANQAGS--GN--------------ITMAT-YNNTGT--------TP--ALVA	338
Azoarcus communis	A-GENITIANAAS-----------------------------GS--------AA--ATI-	344
Thauera humireducens	N-GENITVANAAS-----------------------------GS--------AT--ATV-	344
Oryzomicrobium terrae	S-GESIAIGNASS-----------------------------GA--------AA--SINV	355
Shewanella atlantica	N-GADVNFAGLAANTTGA--------------LAITN-IAADGT-------ASATS----	337
Nereida ignava	D-GDDILIENFSDTGTATNL-------------TVEA-GNFDGT-------SWET--GVD	341
Desulfofustis glycolicus	E-GHNIAISEFSYLGDSGDA----------ASLDFTT-LNADGE-------DPKATAGPD	342
Nitrosomonas eutropha	D-GRDIRILDFNNSGTTKTI-------------DVKG-IN-----------------GVA	342
Nitrosococcus watsoni	Q-GEDILISEANNTGASGNA------------FNVG----------------G------V	335
Marinobacter salarius	Q-GKDITIEDFTAAGSQQLA------------VQGSG-DP------------S------A	347
Desulfobulbus propionicus	D-GKDIVLANFTHSAATTTI------------DVKSA-DN------------Q------T	340
Nitrospirae bacterium CG 4 9 14 0 8	N-GADIAIQDVLIASTG--------------TLKFTG-DNSDAT-------SQ-------	351
Rhodobacter CACIA14H1	T-GKNIEITDYTAADSGAAAE---------ATMDVTG-LNTDGS-------EDATP---D	337
Cereibacter sphaeroides	N-GDDIMLTSVSSTT---AD------------FKVTA-LKSDGT-------ATATN----	340
Roseovarius mucosus	T-GADILITDFTTGT---GA----------STMDVTA-QNADGT-------ASGATAGTH	340
Bradimonadales bacterium 2099667	D-GRNIQFVVGEQTTGLSVGS--FS-NGFRAAFNHADNEVNDANLNRIF--TAGSY---F	364
Myxococcales bacterium 2026763	D-GRNIEVNVS--DAAATAVT-GLT-S-----------GVSTGSLEF----SSGEQ---F	346
Bordetella SCN 67-23	D-GRNVTIGAL--TLGTAVAT------T-LSDFGLAAAGTTGASLNV----SYKAP---T	344
Legionella pneumophila	D-GRNITVTES--GTGFTAGTDGLT-VT-GGAFD----GALRGTLSI----SA-------	347
Geothermobacter ehrlichii	D-GRNIVVEES--GTGFTTGTDGIN-NG-SGDFAAAASDTLRGKIEL----SA-------	353

Aeromonas hydrophila	L-----LPDVTPTTYIGQIELHSSGDKYSNIDMQGA------------------------	385
Maioricimonas rarisocia	AIA---TIGANTFTGVGNVV-TATSGVT---E-------GLVITIG-----ADP-ADS--	316
Fuerstiella marisgermanici	IVG---TIGGTSFNGSGNLL-TADSGAA---K-------GLSIQTA-----ASA-TDA--	313
Califigura coniformis	VVG---TIGGTSFTGKGNVL-TATSGNA---N-------GLKVELN-----AGTAATA--	317
Mariniblastus fucicola	VAG---TIDGVAAAGSGATL-TATSGAS---K-------GLAVKVG-----ADA-ADA--	317
Stieleria neptunia	IAG---EIDGVAASGKGDTL-TSVSGSS---K-------GTVVRIG-----DLA-TDA--	314
Desulfurivibrio alkaliphilus	VYD---G-------EMGDTVLAR-------------------------------------	355
Acidihalobacter ferooxydans	LYT---S--------VGGTIQVSGAAAE--LA-------TFGFTTS-----GSGSTSA--	362
Thioalkalivibrio sulfidiphilus	LAT---N--------VGQDILVATTGSA---D-------DLNITAG-----GTDRA----	360
Caldimicrobium thiodismutans	---SSQTSTTAHAIKVGRLYIANDRDFK--IS--GLSATQFGT------AEIGSNTNS--	387
Thermodesulfobacterium commune	TIQSQTSATTASAVKVGDLYIANDKNFT--LDLGSVTNTALGN------FEIGSTTGS--	401
Desulfobacterium atlanticum	---DDT-NTTGYIHSIGTITLESPEAIT--ISATGGIDADLGITTG--T-----------	393
Hydrogenoviriga calditoris	---GLADTQKAYAVKVGELQIYGTDSFV--VNES-GIDIVGGSG-PDTS--I----TA--	409
Aquifex pyrophilus	---NNT-NTQASAIKVGDLRIIDDESYT--YDFT-GIAGAFGLTSPSGTVGITD------	398
Persephonella hidrogeniphila	---SGG-N-TGSAVKVGDLTVLSAESFA--YDFT-GVSSPTG------GLGIAATGTA--	398
Thermovibrio guaymasensis	---SGG---TGSAMKVGDLKVLSSNSFG--WDVT-GIEAGLG---------VSAVSTS--	397
Algisphaera agarilytica	I--NRDEGADVLSLVNGNLALGDGLDIT--LNTN-TINLGL-------------------	323
Mucisphaera calidilacus	T--LRDDGQDVLAIVNGNLALGRGTSVS--LNTA-FLSIEL-------------------	320
Aquispirillum sp. LM1	TLSVGGATANRNSTVGGRVLFTSDSAYT--LETS-LASATGGLL--N--V----SATG--	429
P. aeruginosa PAO1	VVAA--GTAATTTIVTGYVQLNSPTAYS--VSGT-GTQAS------Q--VFGNASAAQ--	383
Magnetoglobus multicellularis	KTLV--SEFLDSILVGGYVEFQSNEPFI--LFSG-TGGRLF----------TSSSSTQIP	401
Zobellela dentrificans	-AVD--AATNMAFISQGYIVLSDSKTDA--GI-----------T-VQ--EFAADGATPAT	393
Acidocella sp. MX-AZ02	VIGG--STGTASALAQGQVQLQSSGAFS--TSGT-ANIGQTTTS----------------	392
Acidovorax sp. NO-1	VLAA--DTVANNSLTSGYVVLDSAKSFS--TDVT-TSN-----------AFTDAG--S--	385
Hylemonalla gracilis	TLTA--DSAGQNAIVSGYLTFDSNKSFV--LSHA-AGGTV---AASN--AYVGADTSS--	397
Massilia yuzhufengensis	LAAA--AADGDTATVGGKVTLSSSSAFT--ASSA-GVGVF---T--A--A--ATTYSS--	397
Gulbenkiania indica	GT-A--TTAGNTSIANGTIVFDSEKSFS--VTDA-GSGLV---L-------GG---SS--	377
Vogesella indigofera	APGA--TAAGATTIANGTVVFDSDKSFS--VTDA-GSGLV---L-------GG---SS--	378
Azoarcus communis	GGTA--TNAGASAVATGQLALDSEKSFS--VAAA-N---T---T--D--FFNATAAAG--	387
Thauera humireducens	GGTS--TAAGATAVATGQLVLDSEKSFS--LAAA-N---T---T--D--FFSATAAAG--	387
Oryzomicrobium terrae	GTQA--VGANQGTTVNGQLTLDSDKSFS--ITTT-ATTAA---T--T--FFTGTTASA--	401
Shewanella atlantica	--NL--ATAAATFTAAAAITLSSPDTYT--VTGG-ATAEISG-EASG--VFAG-------	380
Nereida ignava	VISA--ATGANDAVVSGVVRMESSQAFT--ISDQ-EAAAADNTAVTG--YFGSGNAGGTA	394
Desulfofustis glycolicus	QDDFDHDTDGTDIVARGQVTFHSNNAYT--IETD-GDTDL-----LA--VENNASEQ---	389
Nitrosomonas eutropha	VELT--GGADDSTRVVGNVDVYSSKGAI--TAAA-ANA--------E--AFVSATATS--	385
Nitrosococcus watsoni	TVED--TGGDDSIVVGGQVSFQSDKSFT--VTSD-DGTN----TVVG--AGGVTSALS--	382
Marinobacter salarius	IELT--SGGANATRVAGELTLDSSVSFA--ATSD-ATLAAG--SVLN--SAQNTAAGS--	396
Desulfobulbus propionicus	TTLT--TGGNDSTRAAGVVEFSSEDSYT--LASS-VAAGAG--SILN--VGAGTSVIS--	389
Nitrospirae bacterium CG 4 9 14 0 8	TLGG--ATSTDSSVVGGTVTLASQKSFS--MSSS-VAAASG--SLVA--AAASTAVGS--	400
Rhodobacter CACIA14H1	TLTL--DNTDNDATVTGQVTFTSTQTFS--VNSA-EIDSGA--SEVG--FFSDTANAA--	386
Cereibacter sphaeroides	VDIG--FGTNKSAGVTGQVDLVSTKSFS--VAAS-VSGSAT----A--HFANANEGS--	386
Roseovarius mucosus	TATL--DDTDNDVAVSGQVELSSTKQFS--VGTD-VVTAGT----V--FFEAAANTS--	386
Bradimonadales bacterium 2099667	SFGGADRGRAIDLTSVGAVEISSADLIL--ISGD-NSADFFGFS----------ETVA-V	410
Myxococcales bacterium 2026763	RVS----------------------------GGQ---VDYLGLT----------GEQI-V	364
Bordetella SCN 67-23	GVTG-------S--------ISSTGAFA--LAGT---ITQT-------------------	365
Legionella pneumophila	-----------------------VDTIA--IGGT---VANIGLS----------ANIS-K	368
Geothermobacter ehrlichii	-----------------------LDTIA--IGGT---QADIGFT----------AAGI-A	374

Aeromonas hydrophila	SLNALGFYQADKKKYT------	401
Maioricimonas rarisocia	TQTVNGA------NGNVSISD-	331
Fuerstiella marisgermanici	TSTGTTV------ASTLSVTD-	328
Califigura coniformis	VSTVTGA------QGSVAVTD-	332
Mariniblastus fucicola	ALSVDGA------QGNVTVTN-	332
Stieleria neptunia	VLTEDGA------QGNVSIQN-	329
Desulfurivibrio alkaliphilus	----------------------	
Acidihalobacter ferooxydans	NFDSNTL------------T--	370
Thioalkalivibrio sulfidiphilus	----------------------	
Caldimicrobium thiodismutans	EFKN------------------	391
Thermodesulfobacterium commune	QFKN------------------	405
Desulfobacterium atlanticum	-F--------------------	394
Hydrogenoviriga calditoris	TLN-------------------	412
Aquifex pyrophilus	----------------------	
Persephonella hidrogeniphila	THES------------------	402
Thermovibrio guaymasensis	ELQK------------------	401
Algisphaera agarilytica	TLTSGAA-QVTGTPYSFTVTGG	344
Mucisphaera calidilacus	ELDETVATTSLNTEYTFDITGG	342
Aquispirillum sp. LM1	-IQIGGV---------------	435
P. aeruginosa PAO1	KSS-------------------	386
Magnetoglobus multicellularis	VLKA------------------	405
Zobellela dentrificans	TLIDTATTKSDD----------	405
Acidocella sp. MX-AZ02	KLDS------------------	396
Acidovorax sp. NO-1	SLQK------------------	389
Hylemonalla gracilis	TLHK------------------	401
Massilia yuzhufengensis	GLSS------------------	401
Gulbenkiania indica	KLQT------------------	381
Vogesella indigofera	TLKT------------------	382
Azoarcus communis	QLQK------------------	391
Thauera humireducens	QLQK------------------	391
Oryzomicrobium terrae	QLQK------------------	405
Shewanella atlantica	----------------------	
Nereida ignava	TLSA------------------	398
Desulfofustis glycolicus	-MN-------------------	391
Nitrosomonas eutropha	SFSA------------------	389
Nitrosococcus watsoni	S---------------------	383
Marinobacter salarius	TPEE------------------	400
Desulfobulbus propionicus	QFEE------------------	393
Nitrospirae bacterium CG 4 9 14 0 8	SLSK------------------	404
Rhodobacter CACIA14H1	DLET------------------	390
Cereibacter sphaeroides	ELSS------------------	390
Roseovarius mucosus	ELSS------------------	390
Bradimonadales bacterium 2099667	V---------------------	411
Myxococcales bacterium 2026763	GV--------------------	366
Bordetella SCN 67-23	----------------------	
Legionella pneumophila	----------------------	
Geothermobacter ehrlichii	K---------------------	375
