# Supplementary material for: An unbroken network of interactions connecting flagellin domains is required for motility in viscous environments
Source: PLoS Pathog. 2023 May 30;19(5):e1010979. doi: 10.1371/journal.ppat.1010979 (PMC10256154; doi:10.1371/journal.ppat.1010979)
Supplement: S1 Table — (PDF) [file ppat.1010979.s013.pdf]

**S1 Table.** Data collection, phasing and refinement statistics for MAD (SeMet) structures

| | Native FliC-D2D3 **(PDB: 8ERM)** | SeMet FliC-D2D3 | | |
|---|---|---|---|---|
| **Data collection** | | | | |
| Space group | P 1 $2_1$ 1 | | P 1 $2_1$ 1 | |
| Cell dimensions | | | | |
| *a*, *b*, *c* (Å) | 53.49, 36.49, 87.45 | | 53.6, 36.5, 87.7 | |
| α, β, γ (°) | 90.00, 97.30, 90.00 | | 90, 97.185, 90 | |
| | | *Peak* | *Inflection* | *Remote* |
| Wavelength | | 0.97929 | 0.97949 | 0.91162 |
| Resolution (Å) | 31.67-1.47 | 36.5 – 1.6 | 36.5 – 2.0 | 36.5 – 2.2 |
| $R_{sym}$ or $R_{merge}$ | 0.091 (0.816) | 0.098 (0.510) | 0.075 (0.166) | 0.109 (0.194) |
| $I$ / σ$I$ | 11.87 (1.52) | 8.9 (2.4) | 14.3 (7.7) | 13.4 (6.3) |
| Completeness (%) | 98.21 (93.99) | 97.7 (95.0) | 90.4 (83.0) | 85.7 (78.0) |
| Redundancy | 6.7 (6.0) | 1.5 (1.6) | 1.6 (1.7) | 1.5 (1.6) |
| | | | | |
| **Refinement** | | | | |
| Resolution (Å) | 86.74-1.47 | | 35.95-1.6 | |
| No. reflections | 53148 | | 44280 | |
| $R_{work}$ / $R_{free}$ | 0.175 / 0.2021 | | 0.2425 / 0.2717 | |
| No. atoms | | | 3001 | |
| Protein | 2739 | | 2661 | |
| Ligand/ion** | 22 | | | |
| Water | 346 | | 340 | |
| *B*-factors | 18.53 | | 19.06 | |
| Protein | 17.51 | | 16.99 | |
| Ligand/ion | 40.17 | | | |
| Water | 25.27 | | 35.2 | |
| R.m.s deviations | | | | |
| Bond lengths (Å) | 0.02 | | 0.02 | |
| Bond angles (°) | 1.94 | | 2.5 | |
| Ramachandran plot | | | | |
| Favored (%) | 99.75 | | | |
| Allowed (%) | 0.25 | | | |
| Disallowed (%) | 0.00 | | | |

*Each structure was determined using data collected from a single crystal. *Values in parentheses are for highest-resolution shell.

**Sulfate ion and glycerol
