# Supplementary material for: An unbroken network of interactions connecting flagellin domains is required for motility in viscous environments
Source: PLoS Pathog. 2023 May 30;19(5):e1010979. doi: 10.1371/journal.ppat.1010979 (PMC10256154; doi:10.1371/journal.ppat.1010979)
Supplement: S2 Table — (PDF) [file ppat.1010979.s014.pdf]

Supplemental table 2. Refinement statistics for cryo-EM map and model of *P. aeruginosa* PAO1 filament

Reconstruction Box size	432x 432
PDB	8SUG
EMDB	EMDB-40765
Pixel size (Å)	0.92
Dose	50 e^-/$Å^2$
Number of particles	29,945
B-factor	-30.9
Resolution 0.143 FSC Map:Map (Å)	4.2
Resolution 0.5 FSC Model:Map (Å)	5
Ramachandran favored (%)	90.1
Ramachandran allowed (%)	9.34
Ramachandran Outliers (%)	0.57
Clash score	25.2
Molprobity score	2.44
Bond angles rmsd (°)	0.873
Bond length RMSD (Å)	0.004
