# Supplementary material for: An unbroken network of interactions connecting flagellin domains is required for motility in viscous environments
Source: PLoS Pathog. 2023 May 30;19(5):e1010979. doi: 10.1371/journal.ppat.1010979 (PMC10256154; doi:10.1371/journal.ppat.1010979)
Supplement: S3 Table — (PDF) [file ppat.1010979.s015.pdf]

Supplemental table 3. Filament length, swimming speed and motile spread of different *P. aeruginosa* strains

P. aeruginosa strain	Filament length [µm] (Q1 – Q3)	Swimming speed [µm/s] (Q1 – Q3)	Area of motile spread [%]
**PAO1**	4.7<br>(2.0 – 5.4)	45<br>(42 – 57)	100<br>(± 5)
**PAK**	4.8<br>(3.8 – 5.6)	36<br>(42 – 49)	20<br>(± 5)

PAO1-Δ*fliC* complement strain	Filament length [µm] (Q1 – Q3)	Swimming speed [µm/s] (Q1 – Q3)	Area of motile spread [%]
FliC$_{WT}$	5.4<br>(4.2 – 6.2)	42<br>(36 – 49)	100<br>(± 6)
FliC-Q277A	5.9<br>(4.1 – 7.1)	42<br>(35 – 49)	68<br>(± 4)
FliC-N358A	6.0<br>(2.9 – 7.3)	41<br>(35 – 47)	56<br>(± 8)
FliC-Y154A	8.0<br>(4.4 – 9.8)	41<br>(36 – 43)	67<br>(± 2)
FliC-Δ141	5.8<br>(3.5 – 6.9)	37<br>(33 – 40)	90<br>(± 6)
FliC-Δ141-142	6.5<br>(4.1 – 7.5)	28<br>(24 – 35)	59<br>(± 11)
FliC-Δ141-143	4.8<br>(2.8 – 5.8)	28<br>(23 – 32)	23<br>(± 2)
FliC-Δ141-144	3.6<br>(1.8 – 4.7)	17<br>(15 – 24)	12<br>(± 2)
FliC-Δ204	5.2<br>(4.1 – 7.2)	39<br>(33 – 43)	75<br>(± 3)
FliC-Δ204-205	3.3<br>(2.6 – 4.7)	22<br>(20 – 27)	32<br>(± 5)
FliC-Δ202Δ207-208	5.1<br>(3.8 – 6.7)	19<br>(16 – 22)	15<br>(± 2)
FliC-Δ249-250	2.6<br>(1.7 – 4.7)	41<br>(38 – 51)	25<br>(± 3)
FliC-Δ267	5.0<br>(3.4 – 6.8)	44<br>(37 – 52)	50<br>(± 3)
FliC-Δ293	9.1<br>(6.3 – 10.6)	46<br>(43 – 52)	52<br>(± 11)
FliC-Δ292-293	9.1<br>(6.8 – 11.0)	47<br>(42 – 50)	23<br>(± 6)
FliC-Δ291-293	2.2<br>(1.8 – 3.6)	30<br>(27 – 37)	19<br>(± 2)
D0$_{PAO1}$D1-2$_{PAK}$	3.0<br>(2.0 – 4.0)	36<br>(30 – 41)	4<br>(± 1)
D0$_{PAK}$D1-3$_{PAO1}$	5<br>(4.0 – 6.5)	46<br>(38 – 53)	85<br>(± 5)
