# Supplementary material for: An unbroken network of interactions connecting flagellin domains is required for motility in viscous environments
Source: PLoS Pathog. 2023 May 30;19(5):e1010979. doi: 10.1371/journal.ppat.1010979 (PMC10256154; doi:10.1371/journal.ppat.1010979)
Supplement: S4 Table — (PDF) [file ppat.1010979.s016.pdf]

Supplemental table S4. Swimming speed of different *P. aeruginosa* strains in increasing concentration of Ficoll 400.

			PAO1$_{wt}$	PAK$_{wt}$	Q277A	N358A	Y154A	Δ141	Δ267	Δ293
**Ficoll 400 concentration**	0%	**Mean**	45	42	42.5	42	42	36.5	44	46
		**SD***	10.3	9.7	10.3	8.4	9	5	9.9	7.4
		P-value		>0.9999	>0.9999	>0.9999	>0.9999	0.1464	>0.9999	>0.9999
	5%	**Mean**	42	36	38	30	34	27	31	37
		**SD**	6	8.5	6.5	7.3	4.8	5.8	5.4	6
		P-value		0.5303	0.0436	>0.9999	0.0003	0.153	>0.9999	>0.9999
	10%	**Mean**	33	21	25	15	23	16	23	22
		**SD**	4.8	5	7.4	3	3.4	3.3	2.8	4.4
		P-value		0.0003	<0.0001	0.0003	<0.0001	<0.0001	<0.0001	<0.0001
	15%	**Mean**	25	15	11	7	12	11	12	8
		**SD**	3.8	6.4	2.4	2.1	2.2	2.2	2.4	2.5
		P-value		<0.0001	<0.0001	<0.0001	<0.0001	<0.0001	<0.0001	<0.0001

*SD – standard deviation
